# LncRNA *LINRIS* stabilizes IGF2BP2 and promotes the aerobic glycolysis in colorectal cancer

**DOI:** 10.1186/s12943-019-1105-0

**Published:** 2019-12-02

**Authors:** Yun Wang, Jia-Huan Lu, Qi-Nian Wu, Ying Jin, De-Shen Wang, Yan-Xing Chen, Jia Liu, Xiao-Jing Luo, Qi Meng, Heng-Ying Pu, Ying-Nan Wang, Pei-Shan Hu, Ze-Xian Liu, Zhao-Lei Zeng, Qi Zhao, Rong Deng, Xiao-Feng Zhu, Huai-Qiang Ju, Rui-Hua Xu

**Affiliations:** 1 State Key Laboratory of Oncology in South China, Collaborative Innovation Center for Cancer Medicine, Sun Yat-sen University Cancer Center, Guangzhou, China; 2 Department of Medical Oncology, Sun Yat-sen University Cancer Center, Guangzhou, China; 3 Department of Anatomical and Cellular Pathology, State Key Laboratory of Translational Oncology, Prince of Wales Hospital, The Chinese University of Hong Kong, Hong Kong, China; 4 Department of Pathology, Sun Yat-sen University Cancer Center, Guangzhou, China; 5 Precision Diagnosis and Treatment for Gastrointestinal Cancer, Chinese Academy of Medical Sciences, Guangzhou, China

**Keywords:** Autophagy, CRC, IGF2BP2, *LINRIS*, MYC

## Abstract

**Background:**

Long noncoding RNAs (lncRNAs) play nonnegligible roles in the epigenetic regulation of cancer cells. This study aimed to identify a specific lncRNA that promotes the colorectal cancer (CRC) progression and could be a potential therapeutic target.

**Methods:**

We screened highly expressed lncRNAs in human CRC samples compared with their matched adjacent normal tissues. The proteins that interact with *LINRIS* (Long Intergenic Noncoding RNA for IGF2BP2 Stability) were confirmed by RNA pull-down and RNA immunoprecipitation (RIP) assays. The proliferation and metabolic alteration of CRC cells with *LINRIS* inhibited were tested in vitro and in vivo*.*

**Results:**

*LINRIS* was upregulated in CRC tissues from patients with poor overall survival (OS), and *LINRIS* inhibition led to the impaired CRC cell line growth. Moreover, knockdown of *LINRIS* resulted in a decreased level of insulin-like growth factor 2 mRNA-binding protein 2 (IGF2BP2), a newly found N^6^-methyladenosine (m^6^A) ‘reader’. *LINRIS* blocked K139 ubiquitination of IGF2BP2, maintaining its stability. This process prevented the degradation of IGF2BP2 through the autophagy-lysosome pathway (ALP). Therefore, knockdown of *LINRIS* attenuated the downstream effects of IGF2BP2, especially MYC-mediated glycolysis in CRC cells. In addition, the transcription of *LINRIS* could be inhibited by GATA3 in CRC cells. In vivo experiments showed that the inhibition of *LINRIS* suppressed the proliferation of tumors in orthotopic models and in patient-derived xenograft (PDX) models.

**Conclusion:**

*LINRIS* is an independent prognostic biomarker for CRC. The *LINRIS*-IGF2BP2-MYC axis promotes the progression of CRC and is a promising therapeutic target.

## Background

Colorectal cancer (CRC) is an aggressive primary intestinal malignancy with the third leading incidence and second highest mortality of all types of cancers worldwide [1]. In China, over 380,000 new cancer cases are projected to be discovered in the colon and rectum annually [2]. Therefore, finding new therapeutic strategies for CRC is of great significance.

Long noncoding RNAs (lncRNAs) are special RNA molecules that are longer than 200 nucleotides long and have no protein-coding potential [3]. As epigenetic regulators in various diseases, including cancers, lncRNAs are involved in biological processes with diverse mechanisms, such as mediating interactions between DNA and proteins, adsorbing microRNAs, and binding to proteins as decoys [4–6]. Accumulating evidence has shown that, just as oncogenes affect the prognosis of patients, some lncRNAs influence the progression and death of cancer cells [7–9], indicating that targeting lncRNAs could be a new approach for CRC treatment.

As another critical epigenetic regulator, the N^6^-methyladenosine (m^6^A) modification has attracted the attention of researchers worldwide [10]. During the biological processes of m^6^A modifications, there are three types of proteins (‘readers’, ‘writers’ and ‘erasers’) that play irreplaceable roles [11–13]. YT521-B homology domain-containing proteins (YTHDFs) are the well-known m^6^A ‘readers’ that participate in the recognition of m^6^A-modified mRNAs [13, 14]. The insulin-like growth factor 2 mRNA-binding protein (IGF2BP) family consists of three members, IGF2BP1–3, which are newly reported m^6^A ‘readers’ [14]. Unlike YTHDFs, which regulate pre-mRNA splicing and facilitate translation, these proteins are responsible for targeted mRNA stability and are associated with thousands of targets, such as *MYC, KRAS* and *MDR1* [15–17]. In brief, IGF2BPs recognize m^6^A-modified mRNAs and maintain their stability by recruiting RNA stabilizers to promote the progression of cancers [14, 18]. However, the biological mechanism of IGF2BP2 in CRC remains largely unclear.

In this study, we found a highly expressed lncRNA called *LINRIS* (Long Intergenic Noncoding RNA for IGF2BP2 Stability) in CRC. *LINRIS* blocked the degradation of IGF2BP2 through the ubiquitination-autophagy pathway. As a consequence, MYC-mediated glycolysis was downregulated, inhibiting the proliferation of CRC cells in vitro and in vivo.

## Methods

### Cell lines and cell culture

All human CRC cell lines described in this article were purchased from the American Type Culture Collection (Manassas, VA, USA). The cells were grown in basic RPMI-1640 medium (1×) or DMEM (Thermo Fisher Scientific, Waltham, MA, USA) supplemented with 10% fetal bovine serum at 37 °C with 5% CO_2_. All cells tested negative for mycoplasma contamination, and this result was verified by short tandem repeat fingerprinting before use.

### Reagents and antibodies

The reagents and antibodies are listed in Additional file 1: Table S1.

### RNA-sequencing (RNA-seq) analysis

With the raw reads from sequencing, we first retained the qualified reads (also known as clean reads) that passed the quality control step by FastQC software. Reads with low base quality, contamination or containing more than 10% N were removed from further analysis. By using STAR [19], clean reads of each sample were then aligned to the GRCh38 human reference genome from GENCODE. Gene and transcript expression were sequentially estimated with RSEM [20]. To compare the lncRNAs, the genes were grouped in terms of “lncRNA”, “non_coding” and “antisense” according to the annotation from GENCODE. Differential expression analysis was performed using DESeq2 [21], and those RNAs with an adjusted *P* value < 0.05 and a fold change > 1.5 were considered differentially expressed genes. In addition, the genes with < 1 fragments per kilobase of transcript per million fragments mapped were removed.

### Lentivirus and plasmid transfection

The expression of *LINRIS* was knocked down by short hairpin RNAs (shRNAs) targeting human *LINRIS* or by a nonspecific oligonucleotide that was ligated into the LV-3 (pGLVH1/GFP + Puro) vector. The lentiviruses were synthesized by OBiO Technology Co., Ltd. (Shanghai, China) and the sequences are listed in Additional file 2: Table S2. HCT116 and DLD-1 cells were transfected with the lentivirus according to the manufacturer’s instructions. To obtain stably transfected cell lines, these cells were treated with puromycin (2–3 μg/mL) for 2 weeks. After the knockdown efficiency was confirmed by quantitative PCR (qPCR) and Western blotting analyses, the cells were used for subsequent experiments.

The expression vectors for 3FLAG-tagged MS2 coat protein (MCP) and MS2-tagged *LINRIS* were provided by OBiO Technology Co., Ltd. (Shanghai, China), and FLAG-tagged expression vectors for full-length IGF2BP2 and site-directed mutants (K77R and K139R) were provided by Kidan BioTechnology Co., Ltd. (Guangzhou, China). The plasmids were transfected into the cells with Lipofectamine 3000 as recommended by the manufacturer.

### Human tissue specimens

Clinical samples were collected from Sun Yat-sen University Cancer Center (Guangzhou, China). All patients were histologically diagnosed with CRC before the operation. Written informed consent was obtained from all patients. The study was approved by the Medical Ethics Committee of Sun Yat-sen University.

### Immunoprecipitation (IP) assay

An anti-IGF2BP2 antibody (1–2 mg per test, Abcam, ab124930) and an anti-FLAG/DYKDDDDK Tag (1–2 mg per test, Cell Signaling Technology, 8146 s) were used in the IP assays, and the proteins were detected by Western blotting with an anti-ubiquitin antibody (1:1000, Cell Signaling Technology, #3933) according to the manufacturer’s instructions.

### RNA pull-down and RNA immunoprecipitation (RIP) assays

Expression vectors for full-length *LINRIS* and its N-terminal (1–570 nt) and C-terminal (571–913 nt) regions used for the in vitro synthesis of RNA were provided by OBiO Technology (Shanghai, China). The lncRNAs were transcribed in vitro using a MEGAscript™ T7 Transcription Kit (Invitrogen, Carlsbad, CA, USA) and were biotinylated with a Pierce RNA 3′ End Desthiobiotinylation Kit (Thermo Fisher Scientific, Waltham, MA, USA) according to the manufacturer’s instructions. The proteins were extracted from HCT116 and DLD-1 cell lines using Pierce IP Lysis Buffer. Then, RNA pull-down assays were performed with a Pierce Magnetic RNA-Protein Pull-Down Kit (Thermo Fisher Scientific, Waltham, MA, USA). Briefly, the biotinylated lncRNAs were captured with streptavidin magnetic beads and incubated with the cell lysates at 4 °C for 6 h. Then, the mixture was washed and eluted. The eluate was subjected to mass spectrometry or Western blotting analysis. RIP assays were performed with a Magna RNA-Binding Protein Immunoprecipitation Kit (Millipore, Bedford, MA, USA) according to the manufacturer’s instructions. The mixture was digested with proteinase K before the immunoprecipitated RNAs were extracted, purified and subjected to qPCR. The RNA levels were normalized to the input RNA levels (10%).

### Extracellular acidification rate (ECAR) and the measurement of intracellular metabolites

ECAR was measured according to the XF Glycolysis Stress Test protocol on a Seahorse XFe24 Extracellular Flux Analyzer (Agilent Technologies, Santa Clara, CA, USA), and ^13^C-labeled intracellular metabolites were identified as previously described [22]. CRC cells (approximately 1 × 10^7^ cells) were incubated with 2 g/L ^13^C-labeled glucose for 2 h. Metabolites were extracted and detected with a liquid chromatography system equipped with a TripleTOF 5600 mass spectrometer (SCIEX, Framingham, MA, USA). The concentration of the ^13^C-labeled metabolites was normalized to the cell number.

### Chromatin immunoprecipitation (ChIP) assays

ChIP assays were performed with a ChIP kit from Merck Millipore (Billerica, MA, USA) according to the manufacturer’s instructions. qPCR analysis was performed to detect the DNA fragments that coimmunoprecipitated with GATA3.

### RNA interference (RNAi)

The small interfering RNAs (siRNAs) used for in vivo treatment were provided by RiboBio (Guangzhou, China) according to the same sequences as the sh-*LINRIS* (sh-1 and sh-2). The resulting constructs were verified by sequencing. siRNAs were injected into the tumors at two or more spots each time.

### In vivo therapeutic study

All female BALB/c nude mice (3–4 weeks old) used in our study were purchased from the Beijing Vital River Laboratory Animal Technology Co., Ltd. and then housed in specific pathogen-free units.

For the orthotopic models, 2 × 10^6^ cells with negative control (NC, sh-NC), sh-1 or sh-2 in 0.5 mL of PBS were subcutaneously injected into the dorsal flank of 2 mice respectively. After the tumors grew up to 1 cm^3^, they were resected and equally divided into small pieces. Then 15 mice were separated into 3 groups (sh-NC, sh-1 and sh-2), of which the tumor pieces were tied to the base of the ceca. The growth of the tumors was monitored every 2 weeks after intraperitoneal injection of D-luciferin with a Xenogen IVIS 100 Bioluminescent Imaging System. All mice were sacrificed 4 weeks after the surgery.

For the PDX models (PDX#1–3), the tumor tissues were obtained from patients receiving surgeries at our cancer center [23]. After taking a biopsy of a small part of the tumor, we conserved the tissue in ice cold culture medium with 5% penicillin and streptomycin. The tissue was separated into several pieces, which were implanted into the dorsal flanks of mice. To investigate the antitumor effects of RNAi in each PDX model (#1 and #2), 15 female BABL/c nude mice were randomly assigned into 3 groups (NC, si-*LINRIS*#1 and #2) 2 weeks later. The RNAi solution (20 nmol) was injected directly into the tumor bodies of si-*LINRIS*#1 and #2 groups twice per week. PDX#3 was used to investigate the combination effect of RNAi and oxaliplatin. Twenty female BALB/c nude mice were randomly assigned into the following 4 groups: control, oxaliplatin (injected into the abdominal cavity, 5 mg/kg twice per week), RNAi (injected directly into the tumor bodies, 20 nmol twice per week), or combined treatment. The tumor volumes were recorded twice weekly. After treatment for approximately 4 weeks, the mice were sacrificed and all the tumors were extracted and weighed. Furthermore, the tumor tissues were embedded in paraffin, sectioned and stained with hematoxylin and eosin (H&E) or immunohistochemically (IHC) stained with antibodies against Ki-67, IGF2BP2 and MYC according to previously reported protocols [24]. Apoptotic cells in situ were also identified by using a Cell Death Detection Kit (Biotool, Houston, TX, USA) for TdT-mediated dUTP nick end labeling (TUNEL) staining according to the manufacturer’s instructions. The animal study was approved by the Institutional Animal Care and Use Committee of Sun Yat-sen University.

### Statistical analysis

All data are presented as the mean ± SD. Student’s paired or unpaired t-tests and chi-square tests were used for the comparison of significant differences between groups with GraphPad Prism software. Correlations between the *LINRIS* levels and *MYC*, *GLUT-1*, *PKM2* and *LDHA* expression were analyzed with Pearson’s correlation analysis. Survival analyses were performed using the Kaplan-Meier method and assessed using the log-rank test with SPSS and MedCalc statistical software. The levels of significance were set at *, representing *P* < 0.05 and **, representing *P* < 0.01.

Additional methods are described in Additional file 3.

## Results

### *LINRIS* is highly expressed in CRC with poor prognosis

**Fig. 1:**
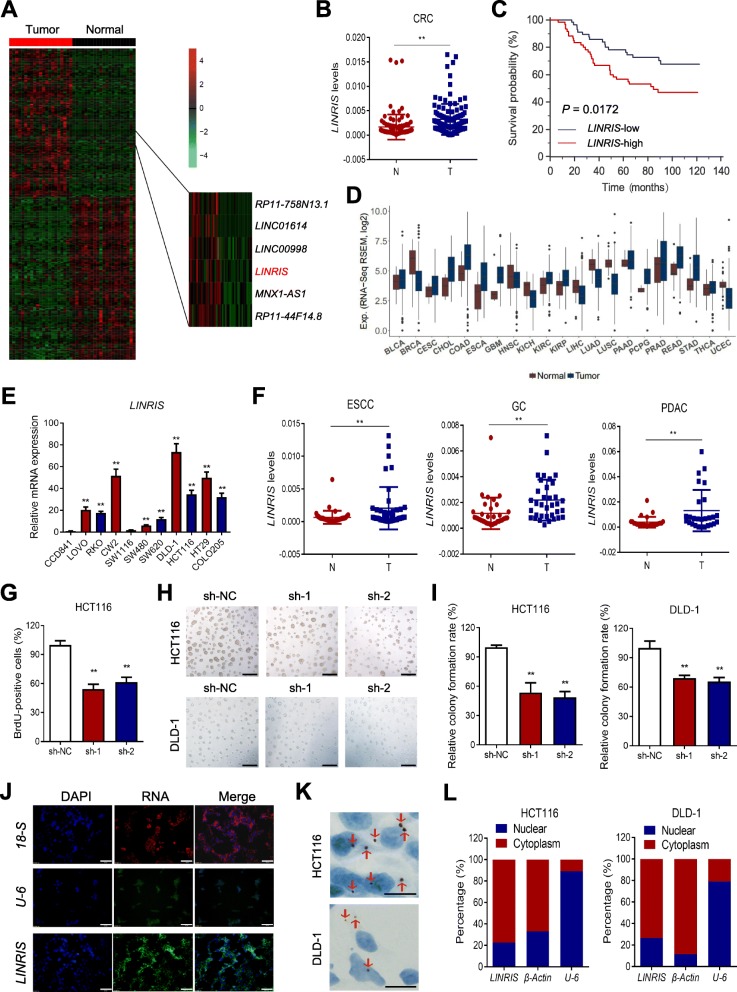
Identification of *LINRIS* as an oncogenic lncRNA in CRC. **a** Heatmap for differentially expressed lncRNAs in CRC tissues compared with that in normal tissues. **b** qPCR detection shows that *LINRIS* was highly expressed in CRC tissues (T, *n* = 118) compared with the expression in normal colon tissues (N). *P < 0.05, **P < 0.01. **c** Kaplan-Meier analysis of the OS curves for CRC patients with *LINRIS*-low (*n* = 58) or *LINRIS*-high (*n* = 60) expression (log-rank test). **d** The overall *LINRIS* expression in multiple human cancers from TCGA. **e** *LINRIS* levels in different CRC cell lines compared with the level in the normal colon cell line CCD841. The data are shown as the mean ± SD; *n* = 3 independent experiments, two-tailed Student’s t-test, *P < 0.05, **P < 0.01. **f** qPCR detection shows that *LINRIS* was highly expressed in ESCC (*n* = 42), GC tissues (*n* = 35) and PDAC (*n* = 27) tissues compared with the expression in normal esophageal, gastric and pancreatic tissues respectively. *P < 0.05, **P < 0.01. **g** BrdU assays of the indicated cells with *LINRIS* knockdown by shRNAs compared with the control. The data are shown as the mean ± SD; n = 3 independent experiments, two-tailed Student’s t-test, *P < 0.05, **P < 0.01. **h** and **i** Images (**h**) and quantification (**i**) of the 3D culture of the indicated cells with or without knocking down *LINRIS* after 1 week. Scale bar, 100 μm. The data are shown as the mean ± SD; n = 3 independent experiments, two-tailed Student’s t-test, *P < 0.05, **P < 0.01. **j** FISH assays identifying the subcellular location of *LINRIS* in HCT116 cells. Scale bar, 100 μm. **k** RNAScope® ISH detection of *LINRIS* expression (arrows) in CRC cells. Scare bar: 20 μm. **l** Location of *LINRIS* in the cytoplasmic and nuclear extractions from CRC cells with qPCR

To exploit lincRNAs that potentially influence the progression of CRC, 21 tumor tissues from patients with stage IV CRC were sent for transcriptome/RNA-seq with paired adjacent normal tissues for comparison. Based on the RNA-seq analysis, we screened out 233 genes coding highly expressed (fold change > 2) lncRNAs (Fig. 1a and Additional file 4: Figure S1A). According to the *P* values and the base mean of these genes, we picked up 30 candidates for further confirmation. Finally, 7 highly expressed lncRNAs were screened for prognosis, including *LINC00920* (renamed as *LINRIS*) (Additional file 4: Figure S1B). Moreover, the RNA-seq analysis showed that NR_046242 (913 bp) was the major transcript of *LINRIS* in CRC (Additional file 4: Figure S1C). We then discovered that higher *LINRIS* expression was correlated with an unfavorable overall survival (OS) of patients with CRC (*n* = 118, clinicopathological features are listed in Additional file 5: Table S3) from Sun Yat-sen University Cancer Center (Fig. 1b and Fig. 1c). In addition, multivariate analysis showed that *LINRIS* was also an independent prognostic factor in the patients with CRC (Additional file 6: Table S4).

According to The Cancer Genome Atlas (TCGA) database, *LINRIS* expression was upregulated in most kinds of tumors and CRC cell lines compared with the expression in normal cells (Fig. 1d and Fig. 1e); these results indicate that *LINRIS* generally acts as an oncogene. Our samples from patients with esophageal squamous cell carcinoma (ESCC), gastric cancer (GC) and pancreas ductal adenocarcinoma (PDAC) also showed the oncogenic status of *LINRIS* in digestive cancers (Fig. 1f). Furthermore, by measuring the expression of *LINRIS* in CRC cell lines and 8 human CRC samples, we found that the increased copy number was account for the upregulation of *LINRIS* in CRC (Fig. 1e and Additional file 4: Figure S1D-S1F). Subsequently, we decided to use two cell lines (HCT116 and DLD-1) with a relatively high *LINRIS* copy number for further research.

Next, we knocked down *LINRIS* in CRC cells with shRNAs (Additional file 4: Figure S1G). BrdU and the 3D-culture assays were used to compare CRC cells transfected with *LINRIS*-specific shRNAs (sh-1 and sh-2) with the nagative control (sh-NC), and the results identified the oncogenic function of *LINRIS* in assisting the growth of cancer cells (Fig. 1g, h, i and Additional file 4: Figure S1H). With RNA FISH assays, we found that *LINRIS* was mainly located in the cytoplasm (Fig. 1j and Additional file 4: Figure S1I), which was further confirmed by the RNAScope® ISH assays and the qPCR analysis of the nuclear and cytosolic extractions (Fig. 1k and l).

### *LINRIS* interacted with IGF2BP2 and maintained its expression

**Fig. 2:**
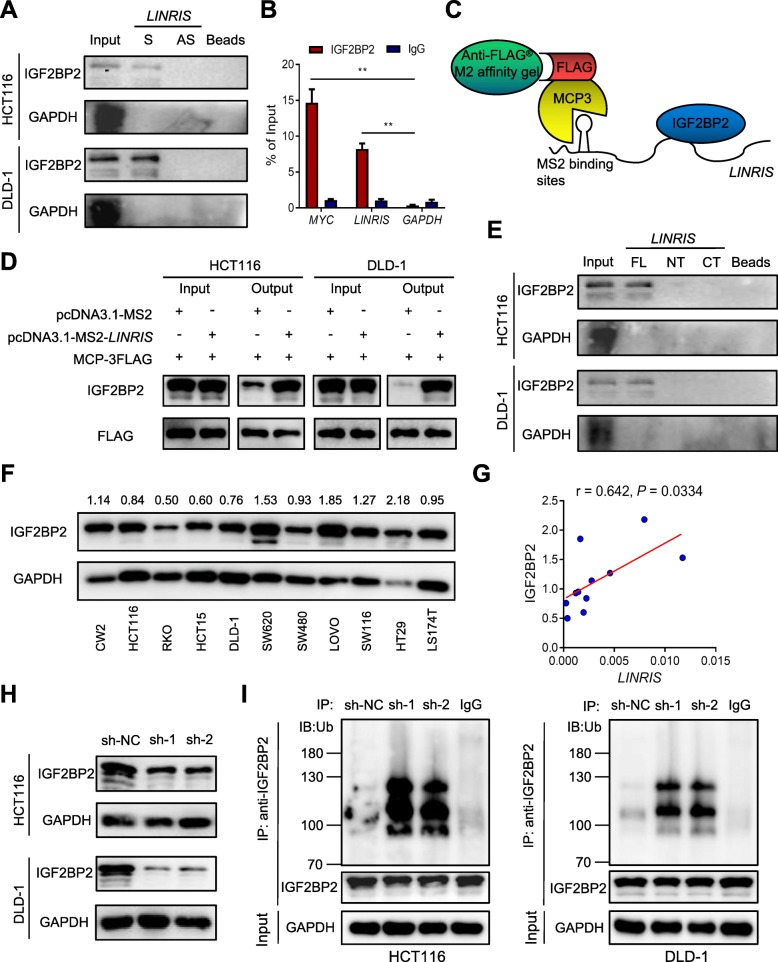
*LINRIS* was associated with IGF2BP2 in CRC. **a** IGF2BP2 was pulled down by biotin-labeled sense *LINRIS* (S) but not *LINRIS* anti-sense (AS) RNA in the indicated cells. **b** RIP assays were applied using anti-IGF2BP2 antibodies with extractions from HCT116 cells. Relative enrichment (mean ± SD) represents the RNA levels associated with the indicated protein relative to an input control from three independent experiments after immunoprecipitation with the anti-IGF2BP2 antibody compared with that with the IgG antibody. *MYC mRNA* was uesd as the positive control and *GAPDH* mRNA was used as the negative control. **c** Expression vectors for FLAG-tagged MCP and MS2-tagged *LINRIS* were transfected into CRC cells to establish the FLAG-MCP-MS2 system. And IGF2BP2 was then pulled down using the anti-FLAG® M2 affinity gel followed by the Western blot analysis. **d** Western blot detection of IGF2BP2 binding to *LINRIS* after FLAG-MCP-MS2 pull-down assays. **e** In vitro-synthesized full-length (FL), N-terminal (NT) and C-terminal (CT) fragments of *LINRIS* were incubated with protein lysates from HCT116 cells. RNA pull-down and Western blotting assays were then performed. The data shown represent three independent experiments. **f** Western blot analysis shows the levels of IGF2BP2 in 11 CRC cell lines with GAPDH as the loading control. **g** IGF2BP2 expression was positively correlated with *LINRIS* expression in CRC cells. The *r* values and *P* values are from Pearson’s correlation analysis. **h** Western blot analysis shows the expression of IGF2BP2 with or without knockdown of *LINRIS* in the indicated cells. **i** CRC cells transfected with shRNAs specific for *LINRIS* or a scrambled control. Cell lysates were immunoprecipitated with either an antibody against IGF2BP2 or an IgG control and then analyzed by immunoblotting with a ubiquitin (Ub)-specific antibody

To identify the molecular mechanism of the *LINRIS*-induced progression of CRC cells, we performed RNA pull-down assays and subsequent mass spectrometry (MS) analysis to explore the proteins that could be potentially associated with *LINRIS* [25, 26]. By comparing independent samples of HCT116 cells with control cells, we mainly identified 18 candidates. Based on the protein score, the protein coverage and the exponentially modified protein abundance index (emPAI) from MS identification (Additional file 7: Figure S2A, B and C), IGF2BP2 was selected as our first target instead of the nuclear proteins such as hnRNPL and HIST1H4A, as it is located mainly in the cytoplasm (Additional file 7: Figure S2D). As shown in Fig. 2a-d, *LINRIS* could directly bind to IGF2BP2. Computational secondary structure analysis of lncRNA was performed with RNAfold (Additional file 7: Figure S2E) [27]. To locate the binding sites of *LINRIS* targeting IGF2BP2, we first constructed two truncated *LINRIS* vectors and performed the RNA pull-down assays, as this lncRNA is only 913 bp in length. However, neither the N-terminal (1–570 nt) nor the C-terminal (571–913 nt) fragments of *LINRIS* could tightly bind to IGF2BP2 (Fig. 2e). Therefore we constructed another vector with 450–640 nt fragments of *LINRIS* according to its secondary structure. The RNA pull-down assays showed that this middle region (450–640 nt) was responsible for the binding between *LINRIS* and IGF2BP2 (Additional file 7: Figure S2F).

By measuring the expression of *LINRIS* and IGF2BP2 in human CRC cell lines (Fig. 2f and Additional file 7: Figure S2G), we found a positive correlation between them (Fig. 2g). Intriguingly, IGF2BP2 was obviously downregulated with *LINRIS* knockdown in both HCT116 and DLD-1 cells (Fig. 2h). Moreover, knocking down *LINRIS* significantly increased the ubiquitination of IGF2BP2 but repressed IGF2BP2-regulated mRNAs (Fig. 2i and Additional file 7: Figure S2H). Therefore, we assumed that IGF2BP2 might be the key to the molecular mechanism of *LINRIS* and that the degradation of IGF2BP2 was probably prevented by this lncRNA.

### *LINRIS* protected IGF2BP2 from autophagic degradation

**Fig. 3:**
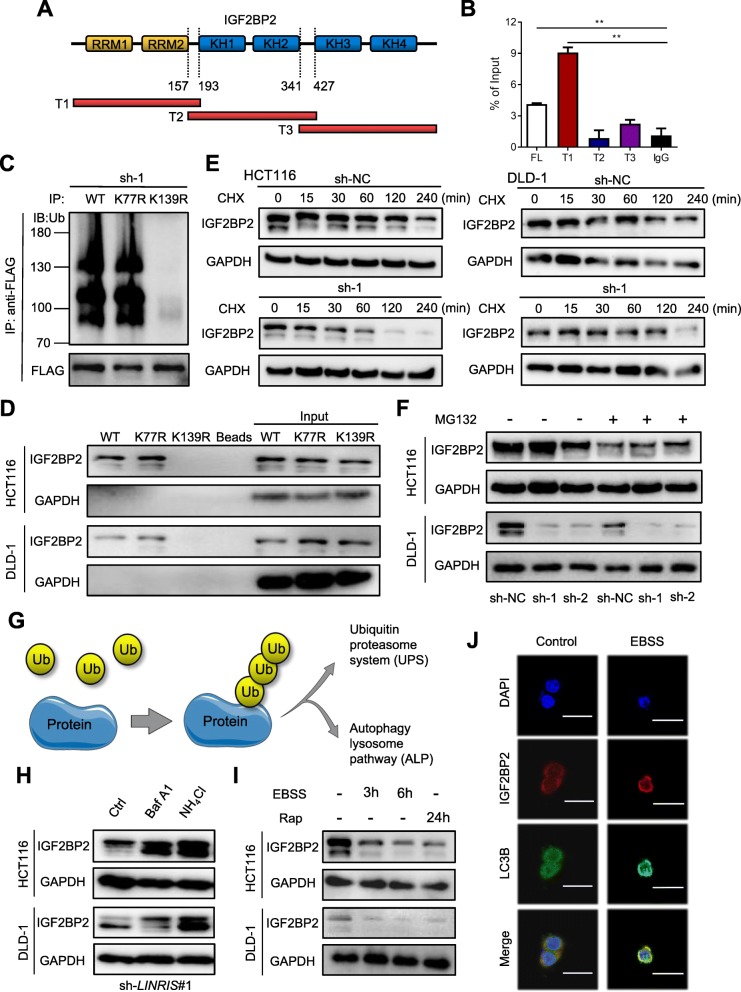
*LINRIS* is involved in the autophagic degradation of IGF2BP2. **a** Schematic structures of IGF2BP2 proteins and three truncated mutants (T1: 1–193; T2: 157–427; T3: 341–913) of IGF2BP variants used in this study. Orange boxes are RRM domains, and blue boxes are KH domains. **b** RIP assays were performed using anti-FLAG antibodies and HCT116 cells transfected with vectors expressing the FLAG-tagged FL and the truncated mutants (T1-T3) of IGF2BP2. The data shown represent three independent experiments. The data are shown as the mean ± SD; *n* = 3 independent experiments, two-tailed Student’s t-test, *P < 0.05, **P < 0.01. **c** IP assays were performed using anti-FLAG antibodies and HCT116 cells transfected with vectors expressing the FLAG-tagged WT or IGF2BP2 mutants (K77R and K139R). **d** Western blot analysis following RNA pull-down assays shows that the K139R mutation of IGF2BP2 blocked its binding to *LINRIS* in CRC cells. GAPDH was used as the loading control. **e** CRC cells with or without shRNAs specific for *LINRIS* were treated with 20 μg/ml CHX or a vehicle for the indicated periods of time. IGF2BP2 levels were analyzed by western blotting. **f** CRC cells with *LINRIS* knockdown and control cells were treated with or without MG132 (5 μM) for 12 h. Cell lysates were analyzed by Western blotting with GAPDH as the loading control. **g** Illustration of the protein degradation process via two main pathways. **h** CRC cells with *LINRIS* knockdown were treated with or without Baf A1 (100 nM, 24 h) and NH_4_Cl (10 mM, 4 h). Cell lysates were analyzed by Western blotting with GAPDH as the loading control. **i** Western blotting shows the levels of IGF2BP2 in the indicated cells after treatment with or without EBSS for 3–6 h or Rap (100 nM) for 24 h. GAPDH was used as the loading control. **j** Confocal microscopy of HCT116 cells treated with or without EBSS for 3 h. Scale bar, 50 μm

IGF2BP2 consists of 2 RNA recognition motifs (RRMs) and 4 K homology (KH) domains and we established three FLAG-tagged vectors containing fragments of IGF2BP2 overlapping with each other on the inactive sections (Fig. 3a). The RIP assay showed that *LINRIS* mainly bound to the region of RRMs (1–157 bp) (Fig. 3b). Then, we obtained the experimentally identified ubiquitination sites of IGF2BP2 from the CPLM databases [28], including two ubiquitinated lysine (K) residues (K77 and K139) in this region. As shown in Fig. 3c, the K139R ubiquitin mutation (unable to form K63-linked chains) in place of K77R significantly impaired the ubiquitination of IGF2BP2 compared with that of the wild-type (WT). In addition, this mutation also abolished the binding between *LINRIS* and IGF2BP2 in RNA pull-down assays (Fig. 3d), suggesting that *LINRIS* suppressed the ubiquitination of IGF2BP2 by masking K139.

To elucidate the degradation pattern of IGF2BP, the CRC cells with downregulated *LINRIS* expression were treated with the protein synthesis inhibitor cycloheximide (CHX) and exhibited a shorter IGF2BP2 half-life than the untreated control cells (Fig. 3e). However, in sh-*LINRIS*-transfected cells, endogenous IGF2BP2 expression could not increase when cells were treated with the proteasome inhibitor MG132 (Fig. 3f), indicating that the degradation of IGF2BP2 may be linked to a more complex mechanism than the ubiquitin-proteasome pathway.

Apart from the ubiquitin-proteasome system (UBS), cellular proteins could be degraded from the autophagy-lysosome pathway (ALP) after ubiquitination (Fig. 3g) [29]. As shown in Fig. 3h and Additional file 8: Figure S3A, the reduction in the endogenous IGF2BP2 protein was successfully reversed by the autophagy inhibitors bafilomycin A1 (Baf A1), NH_4_Cl and 3-methyladenine (3-MA). In contrast, the autophagy activators Earle’s balanced salt solution (EBSS) and Rapamycin (Rap) decreased the levels of IGF2BP2 (Fig. 3i). EBSS-treated cells also exhibited an increased colocalization of IGF2BP2 and LC3B (Fig. 3j and Additional file 8: Figure S3B). Furthermore, we used small guide RNAs (sgRNAs) against autophagy-related gene 5 (ATG5) in DLD-1 cells to block the autophagy system (Additional file 8: Figure S3C). As shown in Additional file 8: Figure S3D, the IGF2BP2 protein levels remained stable when *LINRIS* was inhibited in ATG5-depleted cells. Overall, the above observation suggests that the *LINRIS*-mediated degradation of IGF2BP2 was realized through the ubiquitination-autophagy pathway.

### MYC-mediated glycolysis was influenced by the interaction between *LINRIS* and IGF2BP2

**Fig. 4:**
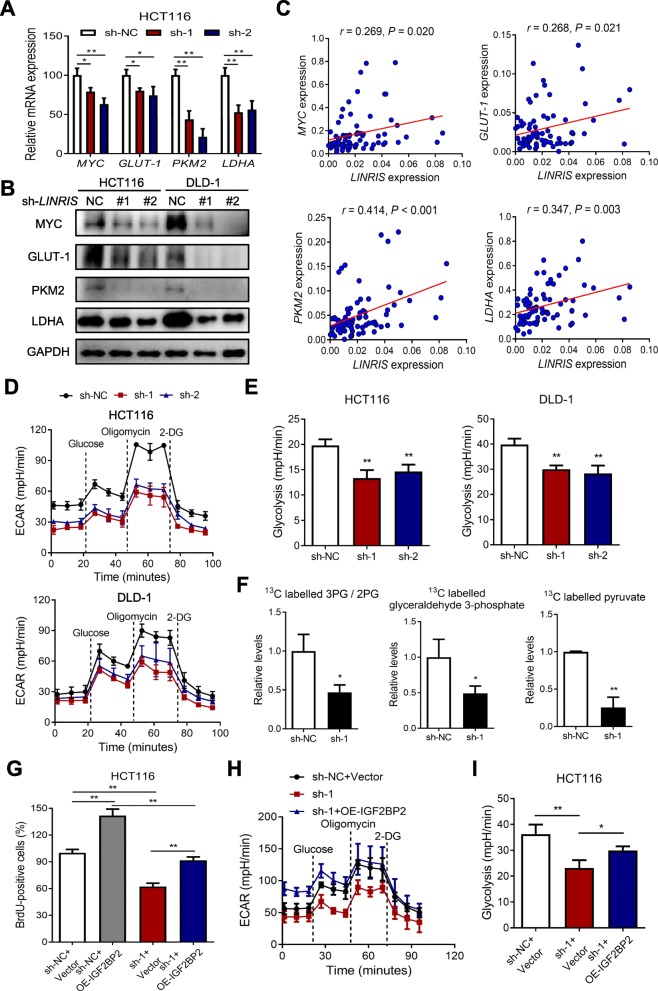
The alteration of *MYC*-mediated glycolysis under the influence of *LINRIS*. **a** The mRNA levels of *MYC* and downstream genes, including *GLUT-1, PKM2* and *LDHA*, when inhibiting *LINRIS* in HCT116 cells. The data are shown as the mean ± SD; n = 3 independent experiments, two-tailed Student’s t-test, *P < 0.05, **P < 0.01. **b** Western blot analysis showed the levels of MYC and downstream genes, including GLUT-1, PKM2 and LDHA, when inhibiting *LINRIS* in CRC cells. **c** Correlations between the *LINRIS* levels and the mRNA levels of *MYC, GLUT-1, HK2, PKM2* and *LDHA* in CRC (*n* = 74). RNA levels were determined by qPCR relative to the levels of GAPDH. The *r* values and *P* values are from Pearson’s correlation analysis. **d** The ECAR was detected in the indicated cells with sh-1, sh-2 or control using an XF Extracellular Flux Analyzer. Glucose, oligomycin and 2-DG were injected sequentially at different time points as indicated. The data shown represent three independent experiments. **e** Statistical analysis of the effects of *LINRIS* knockdown on glycolytic activity. The data are shown as the mean ± SD; n = 3 independent experiments, two-tailed Student’s t-test, *P < 0.05, **P < 0.01. **f** Statistical analysis of ^13^C-labeled 3-PG/2-PG, ^13^C-labeled glyceraldehyde 3-phosphate and ^13^C-labeled pyruvate. The data are shown as the mean ± SD; n = 3 independent experiments, two-tailed Student’s t-test, *P < 0.05. **g** BrdU assay showing that the overexpression (OE) of IGF2BP2 rescued the proliferation inhibition of the indicated cells with the knockdown of *LINRIS*. The data are shown as the mean ± SD; n = 3 independent experiments, two-tailed Student’s t-test, *P < 0.05, **P < 0.01. **h** The extracellular acidification rate (ECAR) was detected in HCT116 cells with or without sh-1 and overexpressed IGF2BP2 using an XF Extracellular Flux Analyzer. Glucose, oligomycin and 2-DG were injected sequentially at different time points as indicated. The data shown represent three independent experiments. **i** Overexpression of IGF2BP2 reversed the suppression of *LINRIS* knockdown on glycolytic activity in HCT116 cells. The data are shown as the mean ± SD; *n* = 3 independent experiments, two-tailed Student’s t-test, *P < 0.05, **P < 0.01

Our group is always eager to explore the metabolic alteration in gastrointestinal cancers [23, 30, 31]. As the mRNA of *MYC* (mainly referred to as *c-Myc*) is recognized by IGF2BP2 as its well-known downstream target [14], we investigated the change in glycolysis during the interaction between *LINRIS* and IGF2BP2. As shown in Fig. 4a, b and Additional file 9: Figure S4A, its downstream genes mediating glycolysis, including *GLUT-1*, *PKM2* and *LDHA,* also exhibited decreased levels in accordance with *MYC* when *LINRIS* was knocked down. In addition, the mRNA levels of *MYC*, *GLUT-1*, *PKM2* and *LDHA* positively correlated with the *LINRIS* levels in CRC tissues (Fig. 4c). As shown in Fig. 4d and e, silencing *LINRIS* resulted in the attenuation of glycolysis in HCT116 and DLD-1 cells. To obtain a deeper view of the metabolic flux of glucose, we incubated CRC cells with ^13^C-labeled glucose before utilizing the liquid chromatography-mass spectrometry (LC-MS). Figure 4f shows that some typical glycolysis productions were reduced with *LINRIS* knockdown. In conclusion, *LINRIS* is essential for maintaining glycolysis in cancer cells, which could influence the proliferation of CRC.

Moreover, we transfected CRC cells with plasmids overexpressing IGF2BP2. As shown in Fig. 4g-i and Additional file 9: Figure S4B-S4E, the impaired proliferation and glycolysis induced by *LINRIS* knockdown was rescued to some extent in HCT116 and DLD-1 cells. In addition, transfecting CRC cells with K139R-mutated IGF2BP2 could fully reverse or even enhance the proliferation impaired by *LINRIS* (Additional file 9: Figure S4F), further confirming the mechanism of the *LINRIS*-IGF2BP2-MYC axis.

### Inhibition of *LINRIS* suppressed CRC growth in vivo

**Fig. 5:**
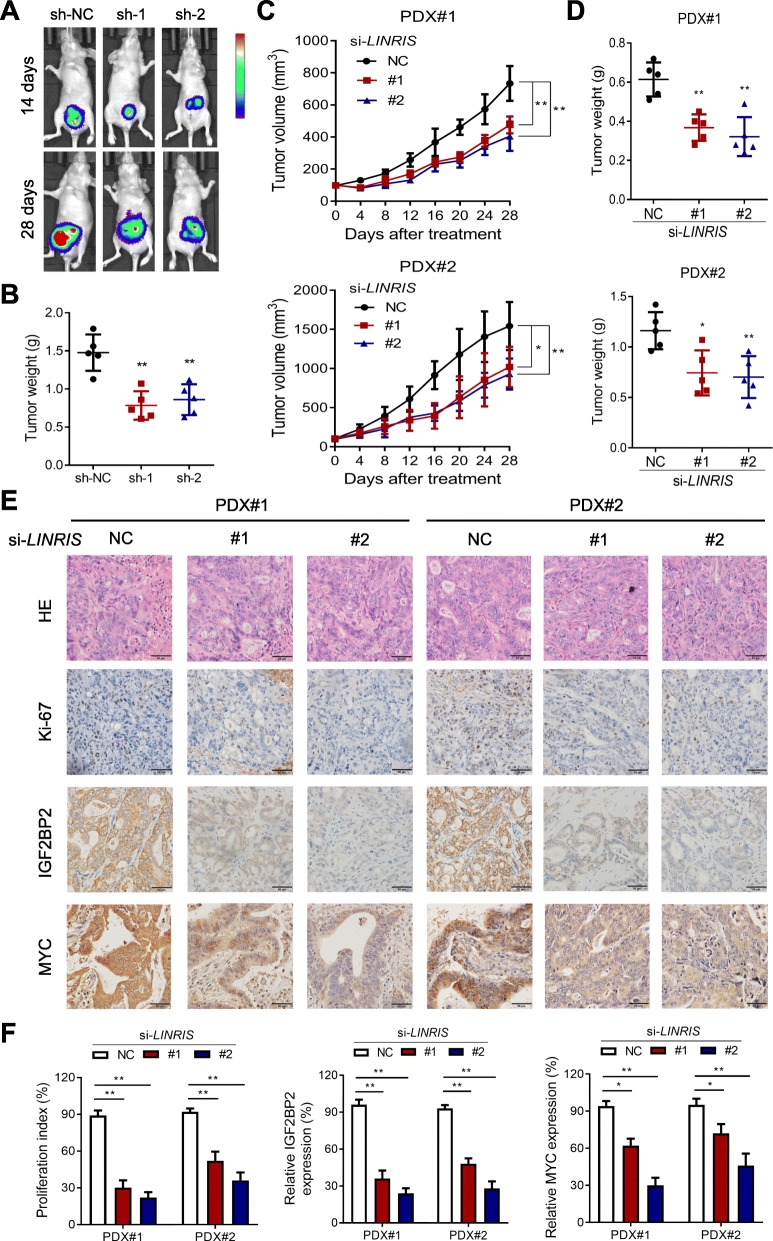
In vivo experiments elucidated the effect of the inhibition of *LINRIS* in CRC. **a** Image of orthotopic tumors with or without *LINRIS* knockdown resected from nude mice. **b** The tumor weights of the orthotopic tumors with or without *LINRIS* knockdown. Error bars, SD of five independent experiments. *P < 0.05 or **P < 0.01 versus the control. **c** and **d** The volume growth curves of tumors (**c**) and the tumor weights (**d**) of two PDX models are shown. Error bars, SD of four independent experiments. *P < 0.05 or **P < 0.01 versus the control. **e** Representative images of H&E staining and IHC staining of Ki-67, IGF2BP2 and MYC from the tumor sections. Scale bar, 50 μm. **f** Quantification of the proliferation index (Ki-67 proportion), IGF2BP2 and MYC levels in the tumor sections. *P < 0.05, **P < 0.01

To investigate whether low *LINRIS* expression would suppress the growth of CRC tumors in vivo, we injected HCT116 cells with or without *LINRIS* knockdown (sh-NC, sh-1 and sh-2) into the ceca of BALB/c nude mice. The HCT116/sh-NC cells formed larger and heavier orthotopic tumors in 4 weeks than did the cells with sh-1 or sh-2 (Fig. 5a and b).

In addition, we tested the in vivo effect of ‘anti-*LINRIS* therapy’ with 2 patient-derived xenograft (PDX) models. As shown in Fig. 5c, d and Additional file 10: Figure S5A, inhibition of *LINRIS* via in vivo-optimized RNA interference (RNAi) significantly suppressed the growth of tumors. Besides, no obvious side effects, such as toxicity or weight loss, were observed (Additional file 10: Figure S5B). IHC staining of the excised tumor sections showed that the expression of Ki-67, which was consistent with that of IGF2BP2 and MYC, decreased with the depletion of *LINRIS* (Fig. 5e and f). Moreover, we explored the clinical perspective of downregulating *LINRIS* in combination with chemotherapy as previous reports suggested [32, 33]. In particular, targeting glycolysis has been reported to be a strategy to overcome chemoresistance [34]. As shown in Additional file 10: Figure S5C and S5D, *LINRIS* inhibition could be performed simultaneously with oxaliplatin treatment. While the proliferation index was decreased, the percentage of apoptotic cells after RNAi and oxaliplatin treatment were higher than the percentage in the control cells (Additional file 10: Figure S5E and S5F). Overall, blocking the *LINRIS*-IGF2BP2-MYC axis is a promising approach for CRC treatment.

### GATA3 inhibited the transcriptional activity of *LINRIS*

**Fig. 6:**
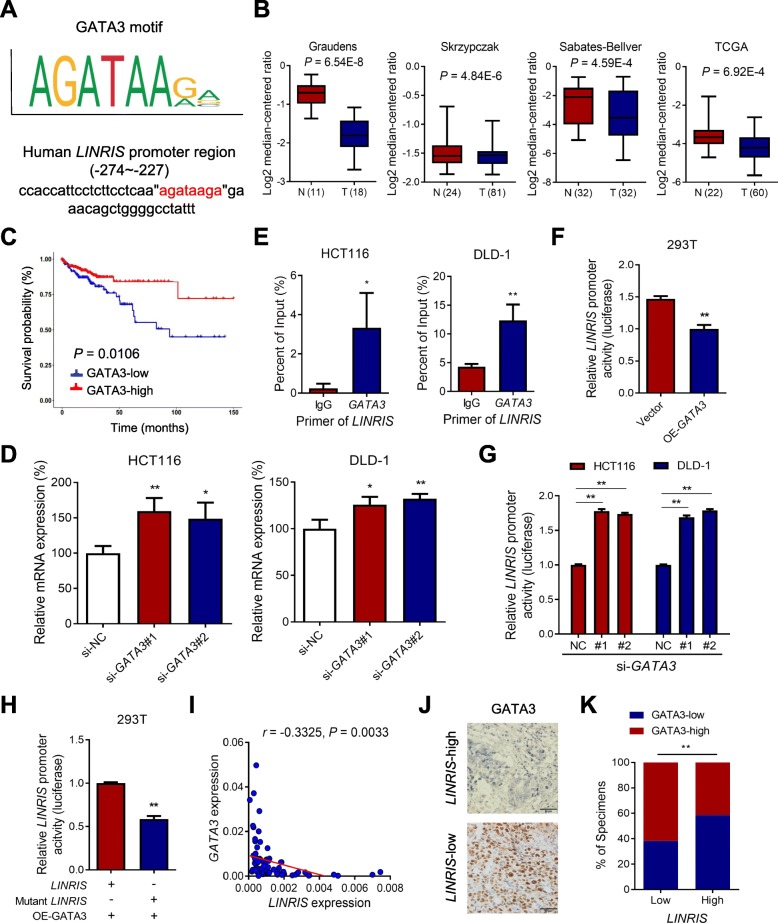
*LINRIS* could be inhibited by GATA3 in CRC. **a** Predicted GATA3 DNA-binding sequences are present in the human *LINRIS* promoter region. **b** *GATA3* expression in several CRC microarray datasets from Oncomine database. **c** Kaplan-Meier analysis of the OS curves for CRC patients with different *GATA3* expression from TCGA database (log-rank test). **d** *LINRIS* mRNA levels were upregulated with knockdown of *GATA3* in CRC cells. The data are shown as the mean ± SD; *n* = 3 independent experiments, two-tailed Student’s t-test, *P < 0.05, **P < 0.01. **e** ChIP-PCR of *LINRIS* in CRC cells. Error bars, SD of three independent experiments. *P < 0.05 or **P < 0.01. **f** Relative *LINRIS* luciferase promoter activity in the indicated cells with GATA3 overexpression (OE-GATA3). Error bars, SD of three independent experiments. *P < 0.05 or **P < 0.01 versus the control. **g** Relative *LINRIS* luciferase promoter activity in the indicated cells with GATA3 knockdown. Error bars, SD of three independent experiments. *P < 0.05 or **P < 0.01 versus the control. **h** Relative *LINRIS* luciferase promoter activity in the indicated cells with or without the mutation of GATA3 binding sequences. Error bars, SD of three independent experiments. *P < 0.05 or **P < 0.01 versus the control. **i** Correlations between the *LINRIS* levels and the mRNA levels of *GATA3* in CRC (*n* = 76). RNA levels were determined by qPCR relative to the levels of GAPDH. The *r* values and *P* values are from Pearson’s correlation analysis. **j** Representative IHC images of GATA3 in tumor tissues from patients with CRC (*n* = 220) with low or high levels of *LINRIS*. Scale bar, 50 μm. **k** Percentages of specimens showing different levels of GATA3 in the low or high *LINRIS* expression groups (n = 220, Chi-square test, **P < 0.01)

To investigate what regulates the transcription of *LINRIS*, we predicted the potential transcription factors with the JASPAR database and found that GATA3 possessed the largest possibility of binding to the promoter of *LINRIS* (Fig. 6a)*.* CRC microarray data from multiple experiments were analyzed with the Oncomine database, which showed that *GATA3* is expressed at lower levels in CRC tissues than in normal tissues (Fig. 6b). Moreover, higher *GATA3* expression is linked with a better prognosis of CRC patients according to TCGA database (Fig. 6c). Inhibition of *GATA3* successfully emancipated the transcription of *LINRIS* (Fig. 6d and Additional file 11: Figure S6A)*.* The ChIP-PCR assays showed that the *LINRIS* promoter region were occupied by GATA3 (Fig. 6e), and the luciferase promoter assays showed that GATA3 overexpression inhibited *LINRIS* activity and vice versa (Fig. 6f and g). Furthermore, the mutation of the GATA3-binding site in the *LINRIS* promoter abolished this transcriptional regulation (Fig. 6h). By analyzing the mRNA expression of *LINRIS* and *GATA3* in human CRC samples (*n* = 76) from Sun Yat-sen Cancer Center*,* we also observed a negative correlation between them (Fig. 6i). We also analyzed another 220 samples from patients with CRC (clinicopathological features are listed in Additional file 12: Table S5) by measuring the expression of *LINRIS* by RNAScope® ISH assays and that of GATA3 with IHC assays (Fig. 6j). As shown in Fig. 6k, increased *LINRIS* expression was more likely accompanied by decreased GATA3 expression and vice versa. In conclusion, GATA3 suppressed the transcription of *LINRIS.*

### The *LINRIS*-IGF2BP2-MYC axis was deeply correlated with the development of CRC

**Fig. 7:**
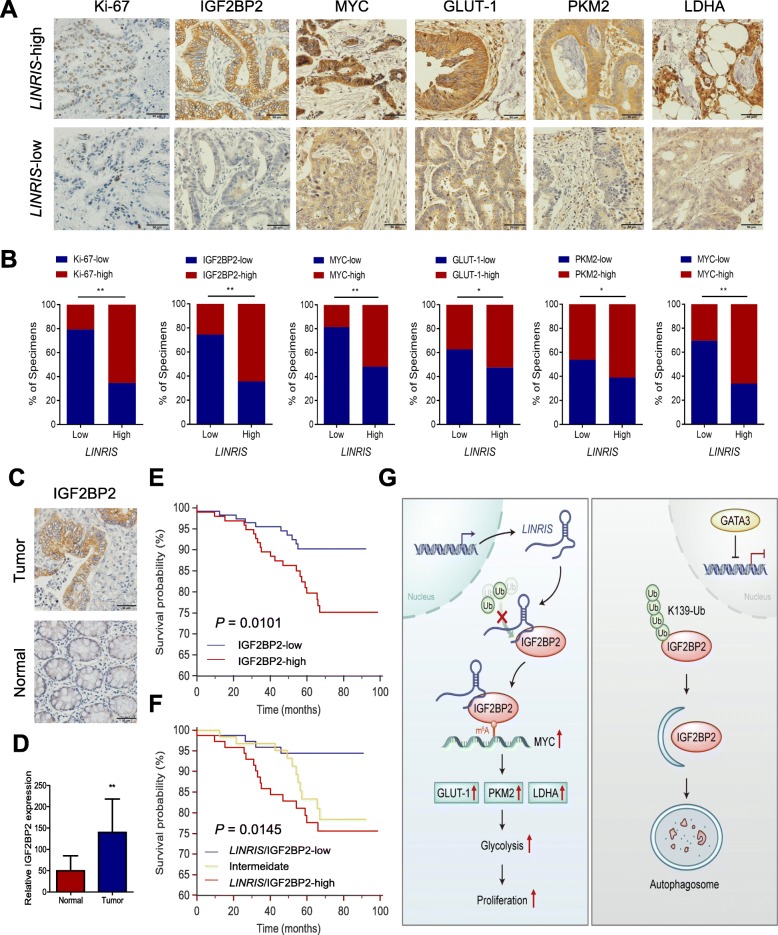
Illustration of *LINRIS*-IGF2BP2-MYC axis in CRC. **a** Representative IHC images of Ki-67, IGF2BP2, MYC, GLUT-1 and PKM2 and LDHA in tumor tissues from patients with CRC (n = 220) with low or high levels of *LINRIS*. Scale bar, 50 μm. **b** Percentages of specimens showing different levels of Ki-67, IGF2BP2, MYC, GLUT-1 and PKM2 and LDHA in the low or high *LINRIS* expression groups (n = 220, Chi-square test, **P < 0.01). **c** and **d** Representative IHC images (**c**) and statistical analysis (**d**) of IGF2BP2 expression in CRC and matched normal tissues. The data are shown as the mean ± SD; n = 220, two-tailed Student’s *t-*test. **e** Kaplan-Meier analysis of the OS curves for CRC patients with low (*n* = 118) or high (*n* = 102) IGF2BP2 expression (log-rank test). **f** Kaplan-Meier analysis of the OS curves for CRC patients with *LINRIS*/IGF2BP2-high (both levels of *LINRIS* and IGF2BP2 were high, n = 76), *LINRIS*/IGF2BP2-low (both levels of *LINRIS* and IGF2BP2 were low, n = 76) or intermediate (*n* = 68) expression (log-rank test). **g** Proposed working model of this study. *LINRIS* stabilized IGF2BP2 by binding to its K139 ubiquitination site and subsequently maintained the MYC-induced glycolysis and proliferation of CRC cells. Otherwise, inhibition of *LINRIS*, such as by GATA3, resulted in more degradation of IGF2BP2 through the ubiquitination-autophagy pathway.

To further illustrate the clinical and pathological significance of the *LINRIS*-IGF2BP2-MYC axis in CRC development, we analyzed 220 samples from the patients described above (Additional file 12: Table S5) by measuring the expression of *LINRIS* by RNAScope® ISH assays, and that of Ki-67, IGF2BP2, MYC, GLUT-1, PKM2 and LDHA with IHC assays (Fig. 7a). As shown in Fig. 7b, the *LINRIS-*high group was consistent with higher expression of Ki-67, IGF2BP2, MYC, GLUT-1, PKM2 and LDHA, whereas *LINRIS-*low group exhibited the opposite outcome.

Moreover, IGF2BP2 expression was significantly higher in the tumor tissues from these patients compared with the matched normal tissues (Fig. 7c and d). Higher IGF2BP2 expression was also associated with a poor prognosis for patients with CRC (Fig. 7e). Then we established a combination scoring system separating the tissues into three groups, namely *LINRIS/*IGF2BP2-high, *LINRIS/*IGF2BP2-low and intermediate. As expected, the *LINRIS/*IGF2BP2-high group showed a poorer prognosis than the other two groups (Fig. 7f, clinicopathological features are listed in Additional file 13: Table S6). In summary, the *LINRIS-*IGF2BP2-MYC axis deeply influenced the development and prognosis of CRC and acts as a potential therapeutic target.

## Discussion

Epigenetic regulation is deeply involved in the genesis and development of cancer cells [35–37]. Among the complex regulatory networks, lncRNAs play a crucial role in affecting the fate of tumors [38–41]. In this study, we used RNA-seq to compare advanced tumors with their paired adjacent normal tissues, and we discovered that *LINRIS* is a highly expressed oncogenic lncRNA that was related to the poor prognosis of patients with CRC. *LINRIS* is located at chromosome 16q21, and few studies have investigated its function or molecular mechanism. We found that downregulating the expression of *LINRIS* resulted in the inhibition of CRC cell proliferation. Moreover, we observed that *LINRIS* interacted with IGF2BP2, whose protein levels were positively correlated with *LINRIS* expression. IGF2BPs recognize m^6^A-modified mRNAs via KH domains and maintain their stability by recruiting RNA stabilizers, such as ELAV-like RNA-binding protein 1 (ELAVL1; also known as HuR), matrin 3 (MATR3) and poly (A)-binding protein cytoplasmic 1 (PABPC1) [14, 42]. Therefore, our findings built a bridge between the epigenetic networks of lncRNAs and m^6^A.

Autophagy is a double-edged sword that determines the survival and death of cells under different circumstances, including interacting with lncRNAs [43–45]. By degrading cellular materials, autophagic degradation is able to change the environmental or nutritional conditions and eliminate damaged organelles [46, 47]. In addition, it is associated with ubiquitination, forming a large degradation system instead of an isolated pathway [29, 48]. In our study, we found that *LINRIS* bound to a ubiquitination site of IGF2BP2, and this binding blocked IGF2BP2 degradation through the ubiquitination-autophagic pathway. Therefore, its downstream mRNAs including *MYC* mRNA were stabilized. To the best of our knowledge, our study is the first to elucidate the degradation pathway of a member of the IGF2BP family.

Because of the Warburg effect, glycolysis is the major method of glucose utilization and determines the progression of cancer cells [49–51]. As *MYC* mRNA is a typical target of IGF2BP2 and one of the core regulators of glycolysis [14, 52, 53], we detected the expression of *MYC* and its downstream enzymes. The downregulation of MYC-related metabolic enzymes resulted in a reduction in glycolysis, which could also account for the proliferation arrest following *LINRIS* knockdown. In contrast, the suppression of cancer cell progression and glycolysis could be reversed by overexpressing IGF2BP2, especially by the K139R mutant without the *LINRIS*-binding site*.*

GATA3 has been discovered to be linked with the development and invasion of cancer cells [54–56]. In this study, we identified GATA3 as a tumor suppressor gene that interacts with *LINRIS.* A decreased GATA3 level was accompanied by upregulation of *LINRIS* and a better prognosis for CRC patients*.* In contrast, overexpression of GATA3 downregulated the transcriptional activity of *LINRIS* in CRC cells.

Furthermore, the in vivo experiments further identified the antitumor effects of inhibiting *LINRIS* in CRC, and the analysis of *LINRIS/*IGF2BP2 expression in the tissues from patients indicated their unique role in the development of CRC; all of these experimental results confirmed the therapeutic potential of targeting the *LINRIS*-IGF2BP2-MYC axis.

## Conclusion

In conclusion, without inhibition factors such as GATA3, *LINRIS* binds to the K139 ubiquitination site of IGF2BP2 and prevents it from degradation via the ALP, maintaining the MYC-mediated glycolysis and the proliferation of CRC cells (Fig. 7g).

## Supplementary information

**Additional file 1: Table S1.** List of reagents and antibodies.

**Additional file 2: Table S2.** The sequences of siRNAs or shRNAs used in this article.

**Additional file 3.** Supplementary methods.

**Additional file 4: Figure S1,** related to Fig. 1. Identification of *LINRIS* as an oncogenic lncRNA in CRC. **(A)** Volcano plots of downregulated and upregulated lncRNA, including *LINRIS*, based on RNA-seq. **(B)** *LINRIS* was highly expressed in CRC tissues (T, *n* = 21) compared with the expression in normal colon tissues (N). *P < 0.05, **P < 0.01. **(C)** RNA-seq analysis shows the expression of *LINRIS* transcripts in CRC tissues. **(D)** In vitro-transcribed *LINRIS* was reverse transcribed and analyzed with qPCR. The standard curve shows that the CT values decreased linearly with increasing *LINRIS* copy number. **(E)** The copy number per cell of *LINRIS* in CRC cell lines compared with CCD841 based on the standard curve of *LINRIS* copy number. The data are shown as the mean ± SD; *n* = 3 independent experiments, two-tailed Student’s t-test, *P < 0.05, **P < 0.01. **(F)** qPCR detection shows the relative RNA levels (left panel) and the copy number per cell (right panel) of *LINRIS* in 8 human CRC samples (P1-P8). The data are shown as the mean ± SD; *n* = 3 independent experiments. **(G)** qPCR detection shows the inhibition of *LINRIS* by shRNAs in the indicated cells. The data are shown as the mean ± SD; n = 3 independent experiments, two-tailed Student’s t-test, *P < 0.05, **P < 0.01. **(H)** BrdU assays of the indicated cells with *LINRIS* knockdown by shRNAs compared with the control. The data are shown as the mean ± SD; n = 3 independent experiments, two-tailed Student’s t-test, *P < 0.05, **P < 0.01. **(I)** FISH assays identifying the subcellular location of *LINRIS* in DLD-1 cells. Scale bar, 100 μm.

**Additional file 5: Table S3.** Correlation between *LINRIS* expression and clinicopathological features in 118 CRC patients.

**Additional file 6: Table S4.** Effect of factors on OS in the CRC patients in the univariate and multivariate Cox regression model.

**Additional file 7: Figure S2,** related to Fig. 2. *LINRIS* was associated with IGF2BP2 in CRC. **(A)** The protein score graph of the 18 proteins identified by the RNA pull-down and LC-MS. **(B)** The emPAI graph of the 18 proteins identified by RNA pull-down assays and LC-MS. **(C)** Protein cover graph of the 18 proteins identified by RNA pull-down assays and LC-MS. **(D)** Immunofluorescence assays identifying the subcellular location of IGF2BP2 in the indicated cells. Bar scale: 50 μm. **(E)** Computational secondary structure of *LINRIS* predicted with RNAfold. **(F)** In vitro-synthesized full-length (FL) and 450–640 nt fragments of *LINRIS* were incubated with protein lysates from HCT116 cells. RNA pull-down and Western blotting assays were then performed. The data shown represent three independent experiments. **(G)** qPCR detection of *LINRIS* levels in 11 CRC cell lines. The data are shown as the mean ± SD; n = 3 independent experiments, two-tailed Student’s t-test, *P < 0.05, **P < 0.01. **(H)** The expression of four representative mRNAs regulated by IGF2BP2 in HCT116 cells with or without sh-*LINRIS*. The data are shown as the mean ± SD; n = 3 independent experiments, two-tailed Student’s t-test, *P < 0.05, **P < 0.01.

**Additional file 8: Figure S3,** related to Fig. 3. *LINRIS* is involved in the autophagic degradation of IGF2BP2**. (A)** Western blotting shows the levels of IGF2BP2 in the indicated cells with the knockdown of *LINRIS* after treatment with or without 3-MA (10 mg/ml) for 24 h. GAPDH was used as the loading control. **(B)** Confocal microscopy of HCT116 cells treated with or without EBSS for 3 h. Scale bar, 50 μm. **(C)** Western blotting shows the knockout of ATG5 in DLD-1 cells with sgRNA (sg-ATG5) compared with that in control cells. GAPDH was used as the loading control. **(D)** DLD-1 cells with the knockout of ATG5 and control cells were transfected with shRNAs specific for *LINRIS*. Cell lysates were analyzed by immunoblotting with GAPDH as the loading control.

**Additional file 9: Figure S4,** related to Fig. 4. The alteration of *MYC*-mediated glycolysis under the influence of *LINRIS*. **(A)** The mRNA levels of *MYC* and downstream genes, including *GLUT-1, PKM2* and *LDHA*, when inhibiting *LINRIS* in DLD-1 cells. The data are shown as the mean ± SD; n = 3 independent experiments, two-tailed Student’s t-test, *P < 0.05, **P < 0.01. **(B)** BrdU assay showing that the overexpression (OE) of IGF2BP2 partially rescued the proliferation inhibition of DLD-1 cells with the knockdown of *LINRIS*. The data are shown as the mean ± SD; n = 3 independent experiments, two-tailed Student’s t-test, *P < 0.05, **P < 0.01. **(C)** The ECAR was detected in DLD-1 cells with or without sh-1 and overexpressed IGF2BP2 using an XF Extracellular Flux Analyzer. Glucose, oligomycin and 2-DG were injected sequentially at different time points as indicated. The data shown represent three independent experiments. **(D)** Overexpression of IGF2BP2 partially reversed the suppression of *LINRIS* knockdown on glycolytic activity in DLD-1 cells. The data are shown as the mean ± SD; n = 3 independent experiments, two-tailed Student’s t-test, *P < 0.05, **P < 0.01. **(E)** BrdU assay showing that the overexpression (OE) of the K139 mutant of IGF2BP2 completely rescued the proliferation inhibition of the indicated cells with knockdown of *LINRIS*. The data are shown as the mean ± SD; n = 3 independent experiments, two-tailed Student’s t-test, *P < 0.05, **P < 0.01.

**Additional file 10: Figure S5,** related to Fig. 5. In vivo experiments elucidated the effect of the inhibition of *LINRIS* in CRC. **(A)** qPCR detection shows the relative RNA levels of *LINRIS* in tumors in two PDX experiments. Error bars, SD of four independent experiments. *P < 0.05 or **P < 0.01 versus the control. **(B)** Curves of the weights of mice treated with RNAi targeting *LINRIS* in two PDX experiments. **(C** and **D)** The volume growth curves of tumors (C) and the tumor weights (D) of PDX#3 are shown. Ctrl, control. Oxa, oxaliplatin. Error bars, SD of five independent experiments. *P < 0.05 or **P < 0.01 versus the control. **(E)** Representative images of H&E staining, immunohistochemistry staining of Ki-67 and TUNEL from the tumor sections. Scale bar, 100 μm. **(F)** Quantification of the proliferation index (Ki-67 proportion) and apoptotic index (TUNEL proportion) in the tumor sections. *P < 0.05, **P < 0.01.

**Additional file 11: Figure S6,** related to Fig. 6. *LINRIS* could be inhibited by GATA3 in CRC. **(A)** qPCR detection shows the inhibition of *GATA3* by siRNA in the indicated cells. The data are shown as the mean ± SD; n = 3 independent experiments, two-tailed Student’s t-test, *P < 0.05, **P < 0.01.

**Additional file 12: Table S5.** Correlation between IGF2BP2 expression and clinicopathological features in 220 CRC patients.

**Additional file 13: Table S6.** Correlation between *LINRIS*/IGF2BP2 expression and clinicopathological features in 220 CRC patients.

## Data Availability

The datasets used and/or analyzed during the current study are available from the corresponding author on reasonable request.

## Abbreviations

3-MA: 3-methyladenine
ALP: Autophagy-lysosome pathway
Baf A1: Bafilomycin A1
ChIP: Chromatin immunoprecipitation
CHX: Cycloheximide
CRC: Colorectal cancer
EBSS: Earle’s balanced salt solution
ECAR: Extracellular acidification rate
emPAI: Exponentially modified protein abundance index
ESCC: Esophageal squamous cell carcinoma
FISH: Fluorescence in situ hybridization
GC: Gastric cancer
H&E: Hematoxylin and eosin
IGF2BP: Insulin-like growth factor 2 mRNA-binding protein
IHC: Immunohistochemically
IP: Immunoprecipitation
KH: K homology
*LINRIS*: Long Intergenic Non-coding RNA for IGF2BP2 Stability
lncRNAs: Long noncoding RNAs
m^6^A: N^6^-methyladenosine
MCP: MS2 coat protein
MS: Mass spectrometry
OS: Overall survival
PDAC: Pancreas ductal adenocarcinoma
PDX: Patient-derived xenograft
qPCR: Quantitative real-time PCR
Rap: Rapamycin
RIP: RNA immunoprecipitation
RNAi: RNA interference
RNA-seq: RNA sequencing
RRMs: RNA recognition motifs
shRNAs: Short hairpin RNAs
siRNAs: Small interfering RNAs
TCGA: The Cancer Genome Atlas
TUNEL: TdT-mediated dUTP nick end labeling
UBS: Ubiquitin-proteasome system
WT: Wild-type
YTHDFs: YT521-B homology domain-containing proteins
