# Supplementary material for: LncRNA LINRIS stabilizes IGF2BP2 and promotes the aerobic glycolysis in colorectal cancer
Source: Mol Cancer. 2019 Dec 2;18:174. doi: 10.1186/s12943-019-1105-0 (PMC6886219; doi:10.1186/s12943-019-1105-0)
Supplement: Supplementary file 1 — Additional file 1: Table S1. List of reagents and antibodies. [file 12943_2019_1105_MOESM1_ESM.docx]

**Table S1** List of reagents and antibodies.

| Name | Manufacturer | Cat. no. |
| --- | --- | --- |
| Cycloheximide | MedChem Express (Shanghai, China) | [HY-12320](https://www.medchemexpress.cn/Cycloheximide.html) |
| bafilomycin A1 | MedChem Express (Shanghai, China) | [HY-100558](https://www.medchemexpress.cn/Bafilomycin_A1.html) |
| NH_4_Cl | Tokyo Chemical Industry (Tokyo, Japan) | A2037 |
| MG-132 | Selleck Chemicals (Houston, TX, USA) | S2619 |
| Oxaliplatin | Selleck Chemicals (Houston, TX, USA) | S1224 |
| Puromycin | Selleck Chemicals (Houston, TX, USA) | S7417 |
| Anti-FLAG^®^ M2 affinity gel | Sigma-Aldrich (St. Louis, MO, USA) | A2220 |
| Rapamycin | Sigma-Aldrich (St. Louis, MO, USA) | 553210 |
| 3-methyladenine | Sigma-Aldrich (St. Louis, MO, USA) | M9281 |
| Fetal bovine serum | Thermo Fisher Scientific (Waltham, MA, USA) | [10438026](https://www.thermofisher.com/order/catalog/product/10438026) |
| Lipofectamine 3000 | Thermo Fisher Scientific (Waltham, MA, USA) | L3000015 |
| Pierce IP Lysis Buffer | Thermo Fisher Scientific (Waltham, MA, USA) | 87788 |
| SuperSignal ECL | Thermo Fisher Scientific (Waltham, MA, USA) | 34577 |
| DAPI | Thermo Fisher Scientific (Waltham, MA, USA) | P36931 |
| Earle's balanced salt solution | Thermo Fisher Scientific (Waltham, MA, USA) | 14155063 |
| TRIzol^®^ reagent | Thermo Fisher Scientific (Waltham, MA, USA) | 15596018 |
| Alexa Fluor^®^ 568 | Thermo Fisher Scientific (Waltham, MA, USA) | R37121 |
| ^13^C-labeled glucose | Cambridge Isotope Laboratories (Andover, MA, USA) | CLM-1396-10 |
| Matrigel | Corning Incorporated (NY, USA) | [356237](https://ecatalog.corning.com/life-sciences/b2c/US/en/Surfaces/Extracellular-Matrices-ECMs/Corning%C2%AE-Matrigel%C2%AE-Matrix/p/356237) |
| Proteinase K | TIANGEN Biotech (Beijing, China) | P4420 |
| D-luciferin | Gold Biotechnology (St Louis, MO, USA) | LUCK-1 |
| Ki-67 nuclear antigen | Zhongshan Golden Bridge Bio-technology (Beijing, China) | ZM-0166-1.5 |
| Triton X-100 | Solarbio (Beijing, China) | T8200 |
| Alexa Fluor^®^ 488 | Cell Signaling Technology (Beverly, MA, USA) | 4412s |
| β-Actin | Cell Signaling Technology (Beverly, MA, USA) | 4970s |
| DYKDDDDK Tag | Cell Signaling Technology (Beverly, MA, USA) | 8146s |
| Ubiquitin | Cell Signaling Technology (Beverly, MA, USA) | #3933 |
| ATG5 | Cell Signaling Technology (Beverly, MA, USA) | 12994s |
| MYC | Cell Signaling Technology (Beverly, MA, USA) | 9402s |
| Rabbit IgG | Cell Signaling Technology (Beverly, MA, USA) | #2729 |
| IGF2BP2 | Abcam (Cambridge, MA, USA) | ab124930/ab128175 |
| GATA3 | Abcam (Cambridge, MA, USA) | ab199428 |
| GAPDH | Abcam (Cambridge, MA, USA) | ab181602 |
| LC3 | Novus Biologicals (Littleton, CO, USA) | NB100-2220 |
| GLUT-1 | HuaAn Biotechnology (Hangzhou, Zhejiang, China) | ET1601-10 |
| PKM2 | HuaAn Biotechnology (Hangzhou, Zhejiang, China) | ER1802-70 |
| LDHA | HuaAn Biotechnology (Hangzhou, Zhejiang, China) | ER00702 |
