# Supplementary material for: LncRNA LINRIS stabilizes IGF2BP2 and promotes the aerobic glycolysis in colorectal cancer
Source: Mol Cancer. 2019 Dec 2;18:174. doi: 10.1186/s12943-019-1105-0 (PMC6886219; doi:10.1186/s12943-019-1105-0)
Supplement: Supplementary file 2 — Additional file 2: Table S2. The sequences of siRNAs or shRNAs used in this article. [file 12943_2019_1105_MOESM2_ESM.docx]

**Table S2** The sequences of siRNA or shRNA used in this article.

| **Names** | **Sequences** |
| --- | --- |
| sh-NC | TTCTCCGAACGTGTCACGT |
| sh-*LINRIS*#1 | CTACATAAAGCAGCCAATA |
| sh-*LINRIS*#2 | CCATTTGGATCAGCTAATA |
| si-*GATA3*#1 | GGCTCTACTACAAGCTTCA |
| si-*GATA3*#1 | GGGCTCTATCACAAAATGA |
