# Supplementary material for: LncRNA LINRIS stabilizes IGF2BP2 and promotes the aerobic glycolysis in colorectal cancer
Source: Mol Cancer. 2019 Dec 2;18:174. doi: 10.1186/s12943-019-1105-0 (PMC6886219; doi:10.1186/s12943-019-1105-0)
Supplement: Supplementary file 3 — Additional file 3. Supplementary methods. [file 12943_2019_1105_MOESM3_ESM.docx]

**Supplementary Methods**

**Quantitative real-time PCR (qPCR)**

Total RNA was extracted from cells by TRIzol^®^ Reagent and then reverse transcribed to cDNA with a Takara kit (Tokyo, Japan). The mRNA expression levels were measured by qRT-PCR in a LightCycler 480 instrument (Roche Diagnostics, Basel, Switzerland) as previously reported. All samples were analyzed in a 10 μL volume system in triplicate. The gene expression was normalized using β-Actin as an internal reference, and the data were analyzed with the 2^-△ct^ method. In addition, the specificity was verified by melting curve analysis. Primers were synthesized by GENEray Biotechnology (Guangzhou, China), and they are listed as following: *LINRIS* (forward: 5’-ACTCTGCCTTTGGCTTTT-3’, reverse: 5’-ACTTTCACTCTTCCCTATGCT-3’); *GATA3* (forward: 5’-TACGCAGCCGACAACCAAATA-3’, reverse: 5’-CTCCGGGAGTCATCCGAGT-3’); *IGF2BP2* (forward: 5’-GCCCCTCATTAAGCCCAAG-3’, reverse: 5’-TTGTGGTGGTCTGACAGTTCG-3’); *MYC* (forward: 5’-GGCTCCTGGCAAAAGGTCA-3’, reverse: 5’-CTGCGTAGTTGTGCTGATGT-3’); *FSCN1* (forward: 5’-CCAGGGTATGGACCTGTCTG-3’, reverse: 5’-GTGTGGGTACGGAAGGCAC-3’); *MARCKSL1* (forward: 5’-CAAGGGTGAAGGGGAGTCG-3’, reverse: 5’-GACAGGCCGCTCAATTTGAAA-3’); *TK1* (forward: 5’-GGGCAGATCCAGGTGATTCTC-3’, reverse: 5’-TGTAGCGAGTGTCTTTGGCATA-3’); *GAPDH* (forward: 5’-GGAGCGAGATCCCTCCAAAAT-3’, reverse: 5’-GGCTGTTGTCATACTTCTCATGG-3’); *β-Actin* (forward: 5’-TGGATCAGCAAGCAGGAGTA-3’, reverse: 5’-TCGGCCACATTGTGAACTTT-3’); *U-6* (forward: 5’-GCTTCGGCAGCACATATACTAA-3’, reverse: 5’-TTGCGTGTCATCCTTGCG-3’); *GLUT-1* (forward: 5’-GGCCAAGAGTGTGCTAAAGAA-3’, reverse: 5’-ACAGCGTTGATGCCAGACAG-3’); *PKM2* (forward: 5’-ATGTCGAAGCCCCATAGTGAA-3’, reverse: 5’-TGGGTGGTGAATCAATGTCCA-3’); *LDHA* (forward: 5’-ATGGCAACTCTAAAGGATCAGC-3’, reverse: 5’-CCAACCCCAACAACTGTAATCT-3’). *LINRIS* promoter (forward: 5’-TTTTGTCTGTTTGCTTACTTCTGAA-3’, reverse: 5’-GCCCAGAGTCTGATCTGAAAGC-3’).

**Cell proliferation and 3D-culture assays**

Cell viability and a BrdU assay were measured using an MTS assay (Qiagen, Hilden, Germany) according to the standard instructions. The absorbance was monitored at a wavelength of 490 nm on a Synergy™ Multi-Mode Microplate Reader (BioTek, Vermont, USA). For the colony formation assay, approximately 10000 cells per well were seeded into 24-well plates on 200 μl Matrigel after transfection with shRNA. After 10 days, the number and size of the cells were observed under the optical microscope, and pictures were taken by CellSens Dimension software.

**Generation of Knockout Cells by CRISPR/Cas9 technology**

ATG5 knockout cells were generated with a CRISPR/Cas9 system [1]. The sequences of small guide RNAs (sgRNAs) targeting ATG5 are as follows: 5’-GTGCTTCGAGATGTGTGGTT-3’.

**Western blot analysis**

GC and CRC cells were trypsinized, washed three times with cold PBS, and resuspended in radioimmunoprecipitation buffer. The samples were centrifuged before lysis for 15 min on ice, and subsequently, the supernatants were collected. After the protein concentrations of the samples were quantified with a BCA kit (Thermo Fisher Scientific, Waltham, MA, USA), the proteins were separated on 8–15% SDS-PAGE gels before being transferred to polyvinylidene fluoride membranes (Immobilon-P, Millipore, Bedford, USA). The membranes were then blocked using 5% nonfat milk dissolved in TBST Q1 for approximately 1 h at room temperature; then, the membranes were incubated with the primary antibody diluted with 5% bovine serum albumin (dissolved in TBST) at 4 °C overnight. Then, the membranes were washed three times with TBST and probed with a peroxidase-linked secondary antibody for 1 h at room temperature before enhanced chemiluminescence reagents (SuperSignal ECL) were added onto the membranes to visualize the proteins.

**Fluorescence in situ hybridization (FISH) and RNAScope® ISH assays**

Oligonucleotide probes complementary to *LINRIS* were purchased from the Exonbio Lab (Guangzhou, China). The probe sequences targeted 105-817 nt of *LINRIS*. These oligonucleotides contain 2’-fluoro-modified RNA residues (2’-F RNA) at bases 3, 6, 15 and 20, which can increase the melting temperature and subsequently enhance the hybridization stability. Both the 5’ and 3’ ends were labeled by digoxin. A scrambled probe (5’-AGTCTATGGTATTCAGTACTCA-3’) was used as a control. Approximately 5-10 μm thick sections from tissues or tissue microarray blocks were deparaffinized, dehydrated and subsequently immersed in 0.2 N HCl for 20 min. Slides were then immersed in 0.5% Tween (PBS) solution, and the tissues were fixed in 10% neutral-buffered formalin. Proteinase K (working solution: 200 μg/ml in PBS) digestion was used to treat fixed tissues at 37 ℃ for 5 min, but culture samples on glass cover slides were treated only with 0.1% Triton-100/PBS. After digestion, slides were immersed in RNase-free water for 3 min and air dried. The slides were then prehybridized in hybridization buffer (65% formamide, 5× SSC, 1% Tween-20, 100 μg/ml yeast RNA) at 37 ℃ for 2 h, followed by hybridization with the probe at 37 ℃ for 24 h. After hybridization, slides were repeatedly washed in 2× SSC with stringent 0.5% Tween-20 for 5 min at room temperature. Finally, an enzyme-labeled fluorescence (ELF) signal amplification kit (Invitrogen, San Diego, CA) was used to amplify and detect lncRNA *in situ* hybridization signals according to the manufacturer’s protocol. The kit used substrate cleavage by a phosphatase to produce a green fluorescence at the site of enzymatic activity. The FISH signal is up to 40 times brighter than those from probes directly labeled with fluorophores. The slides were counterstained with DAPI (Prolong^TM^ Gold Antifade Mountant with DAPI). The images of lncRNA signals in cells were captured by an Olympus FV1000 fluorescence microscope. To make the FISH signals comparable among images, the exposure time for all images captured in the present study was 0.1 second. RNAScope^®^ ISH assays were performed with an RNAScope® 2.0 High Definition Assay Kit (Advanced Cell Diagnostics, Newark, CA, USA) according to the manuscript’s instructions.

**Immunofluorescence assays**

HCT116 and DLD-1 cells were seeded on Glass Bottom culture dishes (Nest Scientific, USA) and incubated for 24 h at 37℃ before fixed with 4% paraformaldehyde for 15 min, and then permeabilized in 0.2% Triton X-100 for 10 min at room temperature. After washing with PBS for 3 times, cells were blocked in 1% bull serum albumin with 0.05% Triton X-100 for 30 min, and then incubated with primary antibodies diluted in 1% bull serum albumin with with 0.05% Triton X-100 for 2 h. The cells were washed, and followed by a fluorescently labeled secondary antibody (Alexa Fluor**^®^** 488- and Alexa Fluor**^®^** 568-conjugated antibodies against mouse or rabbit). Confocal images were performed using a microscope (LSM710; Carl Zeiss) equipped with 100× 1.40 NA oil objectives, with Immersol 518F (Carl Zeiss) as imaging medium and a camera (AxioCam HRc; Carl Zeiss) under the control of Zen 2008 software (Carl Zeiss). The images were processed for gamma adjustments using LSM Zen 2008 or ImageJ software (National Institutes of Health).

**Reporter assays**

The dual reporter expressing Gaussia luciferase under the human *LINRIS* promoter and secreted alkaline phosphatase (SEAP) under the CMV promoter (used for transfection normalization) were established by GeneCopoeia (Rockville, Montgomery, USA). The GATA3-binding site (‘agataaga’) was replaced by ‘TCTATTCT’ in the mutant *LINRIS* promoter. The indicated cells were plated 18 h before transiently transfected with 500 ng of the reporter plasmids using Lipofectamine 3000. 24 h later, plasmids containing GATA3 open reading frame were transfected with Lipofectamine 3000. The luciferase activity was determined according to the manufacturer's instructions (GeneCopoeia, Inc., cat. no. LF032) and normalized to that of the SEAP activity.

**Immunohistochemistry (IHC) assays**

IHC analysis were conducted with standard procedures as previously described [2]. The degree of immunostaining of paraffin-embedded sections was reviewed and scored independently by two pathologists. The scores of staining intensity ranged from 0-3 (0, negative staining; 1, weak staining; 2, moderate staining; 3, strong staining), and a total score was obtained by multiplying the scores for staining intensity and the percentage of cells with positive staining in each microscopic field of view.

**References**

1. Chen M, Meng Q, Qin Y, Liang P, Tan P, He L, Zhou Y, Chen Y, Huang J, Wang RF, Cui J: **TRIM14 Inhibits cGAS Degradation Mediated by Selective Autophagy Receptor p62 to Promote Innate Immune Responses.** *Mol Cell* 2016, **64:**105-119.

2. Wu QN, Liao YF, Lu YX, Wang Y, Lu JH, Zeng ZL, Huang QT, Sheng H, Yun JP, Xie D, et al: **Pharmacological inhibition of DUSP6 suppresses gastric cancer growth and metastasis and overcomes cisplatin resistance.** *Cancer Lett* 2018, **412:**243-255.
