# Supplementary figures and images for: LncRNA LINRIS stabilizes IGF2BP2 and promotes the aerobic glycolysis in colorectal cancer
Source: Mol Cancer. 2019 Dec 2;18:174. doi: 10.1186/s12943-019-1105-0 (PMC6886219; doi:10.1186/s12943-019-1105-0)

**Figure S1**

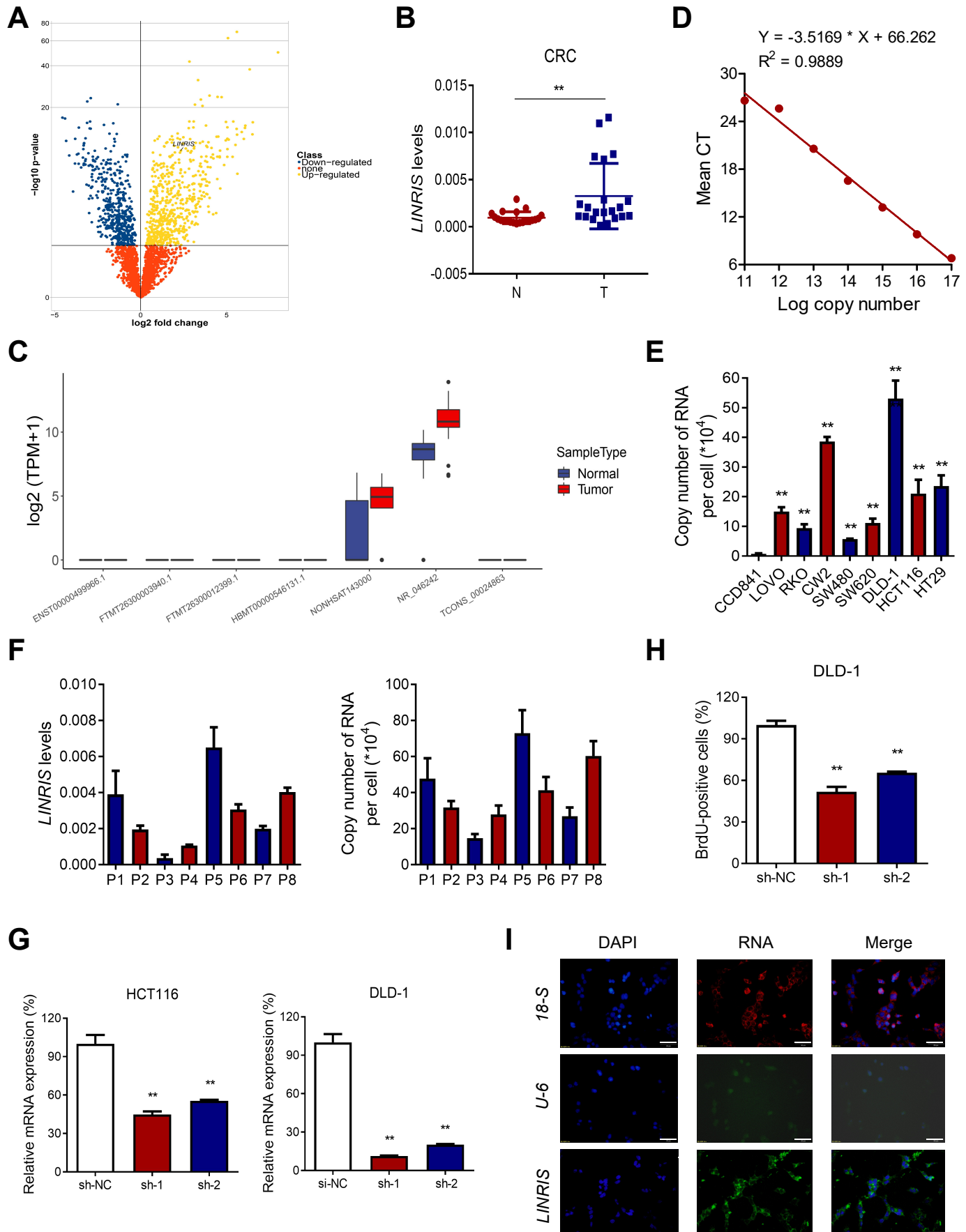

Supplement: Supplementary file 4 — Additional file 4: Figure S1, related to Fig. 1. Identification of LINRIS as an oncogenic lncRNA in CRC. (A) Volcano plots of downregulated and upregulated lncRNA, including LINRIS, based on RNA-seq. (B) LINRIS was highly expressed in CRC tissues (T, n = 21) compared with the expression in normal colon tissues (N). *P < 0.05, **P < 0.01. (C) RNA-seq analysis shows the expression of LINRIS transcripts in CRC tissues. (D) In vitro-transcribed LINRIS was reverse transcribed and analyzed with qPCR. The standard curve shows that the CT values decreased linearly with increasing LINRIS copy number. (E) The copy number per cell of LINRIS in CRC cell lines compared with CCD841 based on the standard curve of LINRIS copy number. The data are shown as the mean ± SD; n = 3 independent experiments, two-tailed Student’s t-test, *P < 0.05, **P < 0.01. (F) qPCR detection shows the relative RNA levels (left panel) and the copy number per cell (right panel) of LINRIS in 8 human CRC samples (P1-P8). The data are shown as the mean ± SD; n = 3 independent experiments. (G) qPCR detection shows the inhibition of LINRIS by shRNAs in the indicated cells. The data are shown as the mean ± SD; n = 3 independent experiments, two-tailed Student’s t-test, *P < 0.05, **P < 0.01. (H) BrdU assays of the indicated cells with LINRIS knockdown by shRNAs compared with the control. The data are shown as the mean ± SD; n = 3 independent experiments, two-tailed Student’s t-test, *P < 0.05, **P < 0.01. (I) FISH assays identifying the subcellular location of LINRIS in DLD-1 cells. Scale bar, 100 μm. [file 12943_2019_1105_MOESM4_ESM.pdf]

**Figure S2**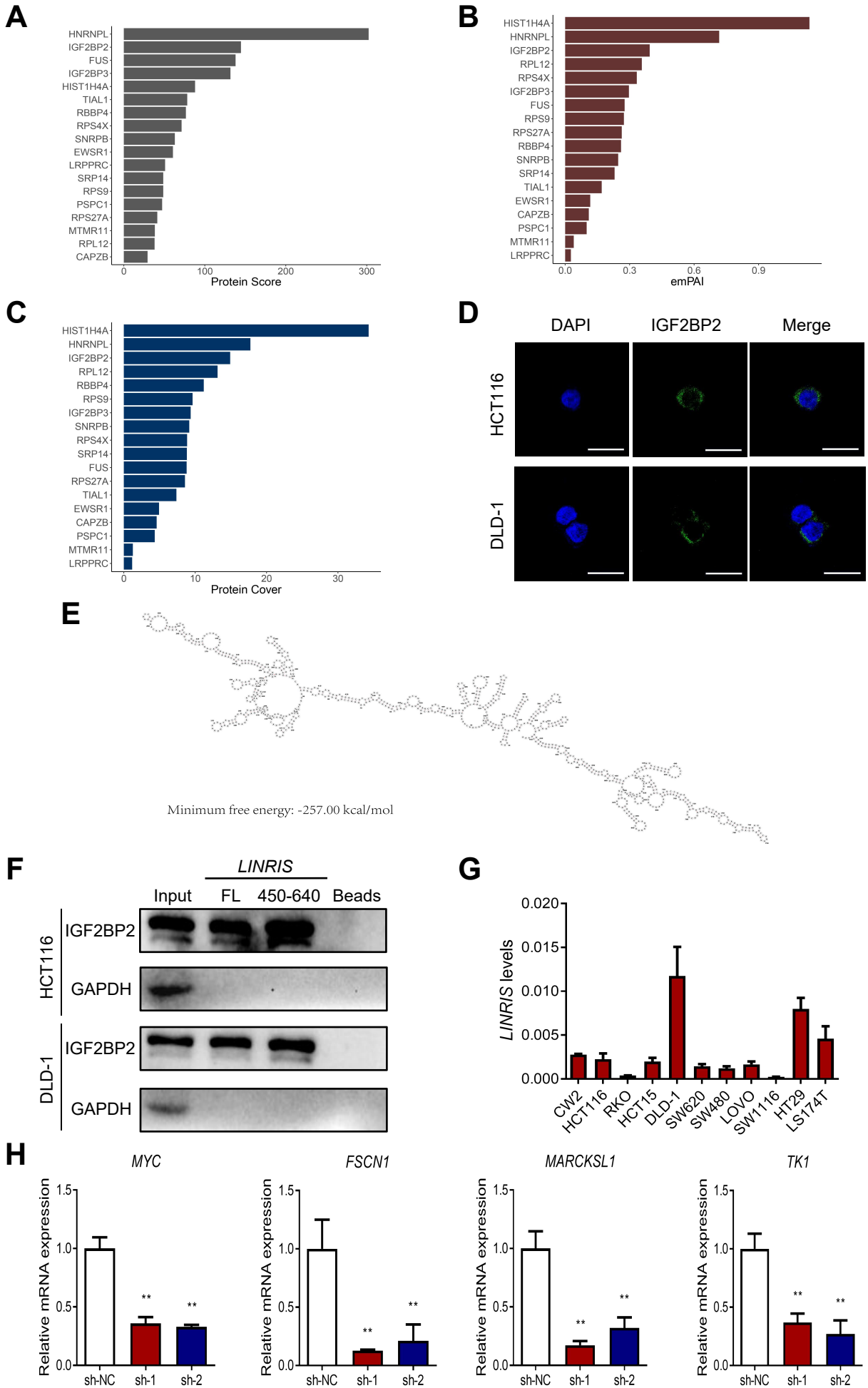

Supplement: Supplementary file 7 — Additional file 7: Figure S2, related to Fig. 2. LINRIS was associated with IGF2BP2 in CRC. (A) The protein score graph of the 18 proteins identified by the RNA pull-down and LC-MS. (B) The emPAI graph of the 18 proteins identified by RNA pull-down assays and LC-MS. (C) Protein cover graph of the 18 proteins identified by RNA pull-down assays and LC-MS. (D) Immunofluorescence assays identifying the subcellular location of IGF2BP2 in the indicated cells. Bar scale: 50 μm. (E) Computational secondary structure of LINRIS predicted with RNAfold. (F) In vitro-synthesized full-length (FL) and 450–640 nt fragments of LINRIS were incubated with protein lysates from HCT116 cells. RNA pull-down and Western blotting assays were then performed. The data shown represent three independent experiments. (G) qPCR detection of LINRIS levels in 11 CRC cell lines. The data are shown as the mean ± SD; n = 3 independent experiments, two-tailed Student’s t-test, *P < 0.05, **P < 0.01. (H) The expression of four representative mRNAs regulated by IGF2BP2 in HCT116 cells with or without sh-LINRIS. The data are shown as the mean ± SD; n = 3 independent experiments, two-tailed Student’s t-test, *P < 0.05, **P < 0.01. [file 12943_2019_1105_MOESM7_ESM.pdf]

Figure S3

A

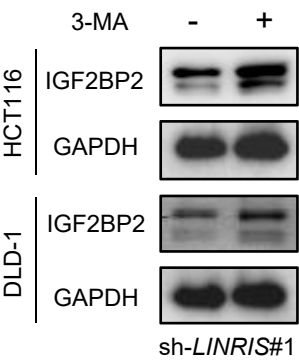

B

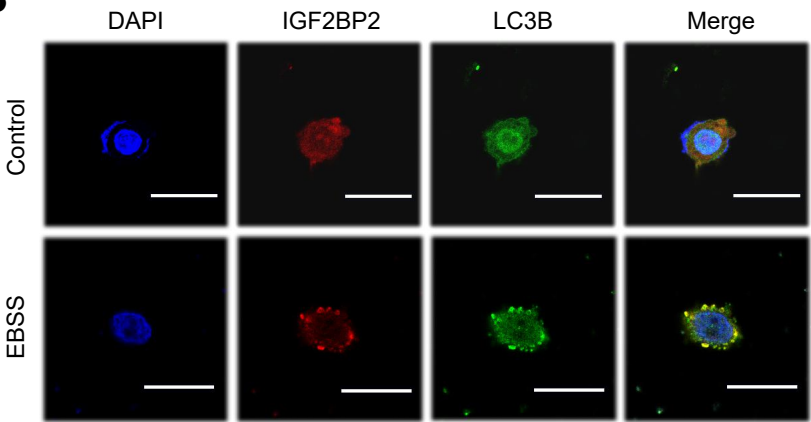

C

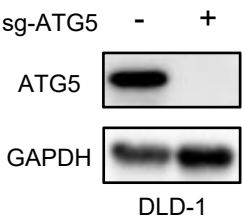

D

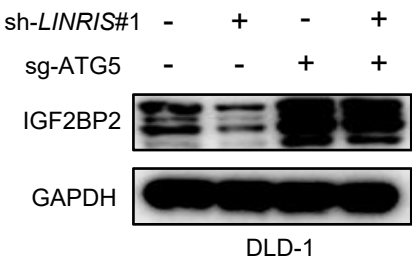

Supplement: Supplementary file 8 — Additional file 8: Figure S3, related to Fig. 3. LINRIS is involved in the autophagic degradation of IGF2BP2. (A) Western blotting shows the levels of IGF2BP2 in the indicated cells with the knockdown of LINRIS after treatment with or without 3-MA (10 mg/ml) for 24 h. GAPDH was used as the loading control. (B) Confocal microscopy of HCT116 cells treated with or without EBSS for 3 h. Scale bar, 50 μm. (C) Western blotting shows the knockout of ATG5 in DLD-1 cells with sgRNA (sg-ATG5) compared with that in control cells. GAPDH was used as the loading control. (D) DLD-1 cells with the knockout of ATG5 and control cells were transfected with shRNAs specific for LINRIS. Cell lysates were analyzed by immunoblotting with GAPDH as the loading control. [file 12943_2019_1105_MOESM8_ESM.pdf]

Figure S4

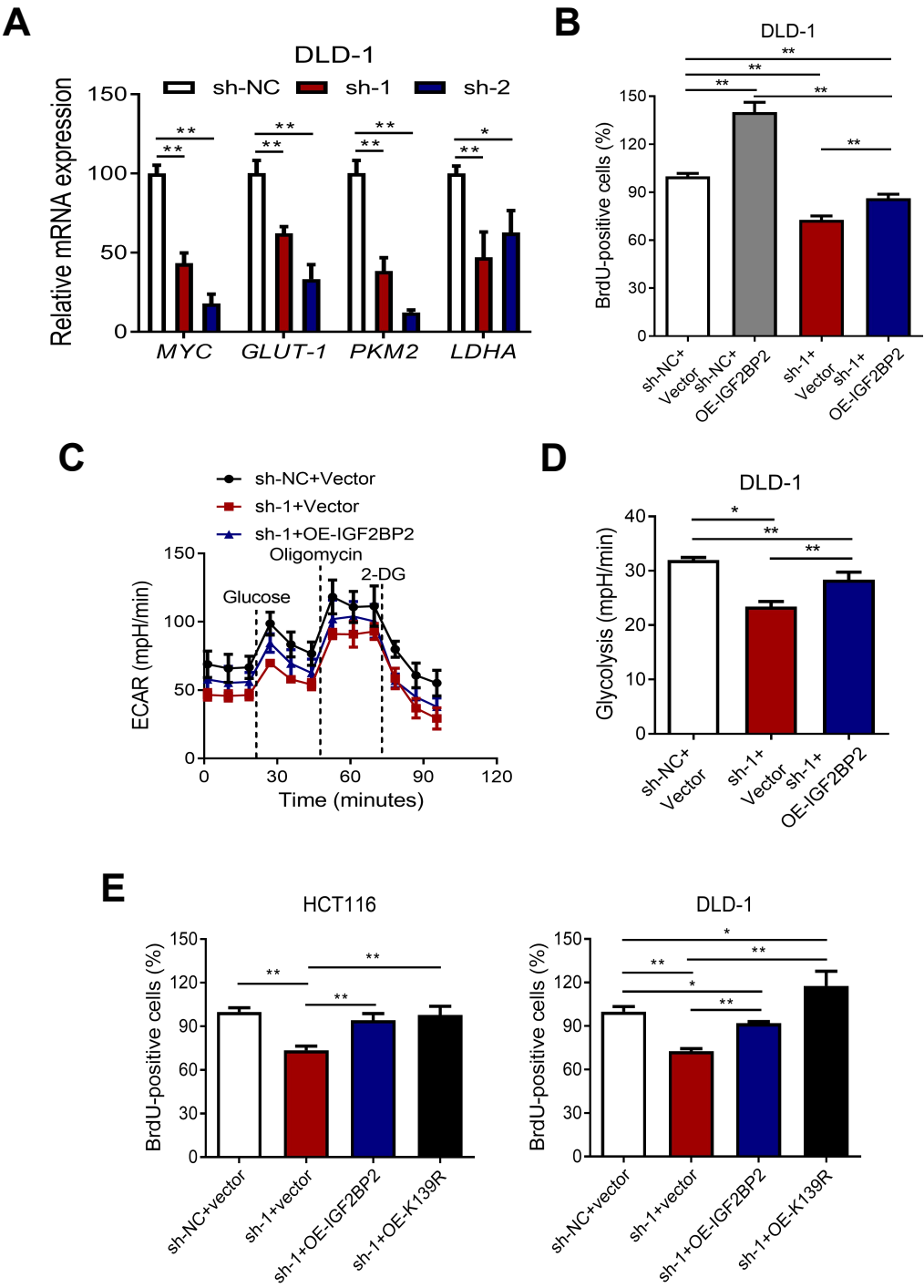

Supplement: Supplementary file 9 — Additional file 9: Figure S4, related to Fig. 4. The alteration of MYC-mediated glycolysis under the influence of LINRIS. (A) The mRNA levels of MYC and downstream genes, including GLUT-1, PKM2 and LDHA, when inhibiting LINRIS in DLD-1 cells. The data are shown as the mean ± SD; n = 3 independent experiments, two-tailed Student’s t-test, *P < 0.05, **P < 0.01. (B) BrdU assay showing that the overexpression (OE) of IGF2BP2 partially rescued the proliferation inhibition of DLD-1 cells with the knockdown of LINRIS. The data are shown as the mean ± SD; n = 3 independent experiments, two-tailed Student’s t-test, *P < 0.05, **P < 0.01. (C) The ECAR was detected in DLD-1 cells with or without sh-1 and overexpressed IGF2BP2 using an XF Extracellular Flux Analyzer. Glucose, oligomycin and 2-DG were injected sequentially at different time points as indicated. The data shown represent three independent experiments. (D) Overexpression of IGF2BP2 partially reversed the suppression of LINRIS knockdown on glycolytic activity in DLD-1 cells. The data are shown as the mean ± SD; n = 3 independent experiments, two-tailed Student’s t-test, *P < 0.05, **P < 0.01. (E) BrdU assay showing that the overexpression (OE) of the K139 mutant of IGF2BP2 completely rescued the proliferation inhibition of the indicated cells with knockdown of LINRIS. The data are shown as the mean ± SD; n = 3 independent experiments, two-tailed Student’s t-test, *P < 0.05, **P < 0.01. [file 12943_2019_1105_MOESM9_ESM.pdf]

**Figure S5**

**A**

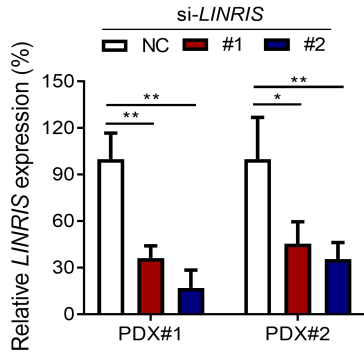

**B**

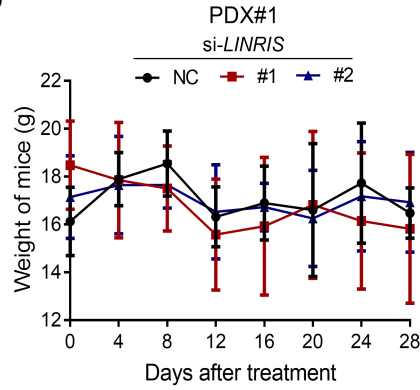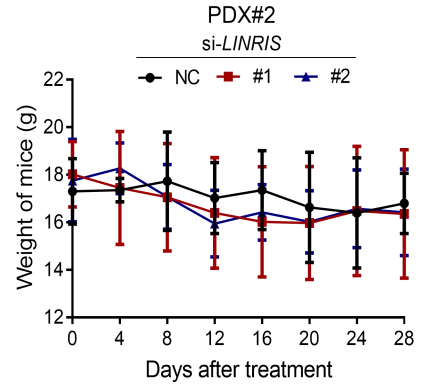

**C**

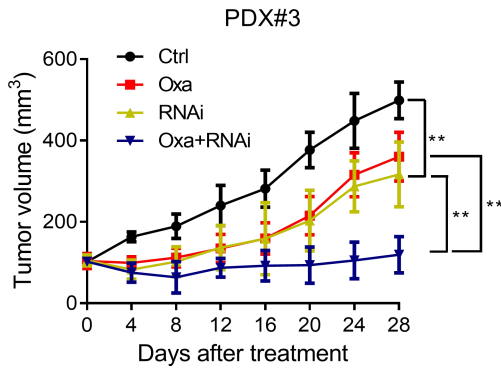

**D**

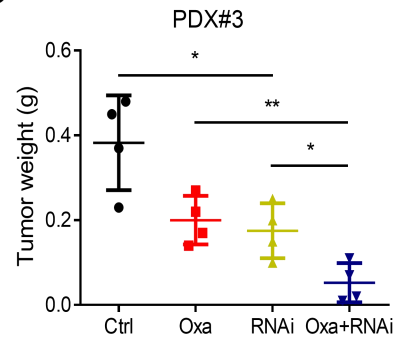

**E**

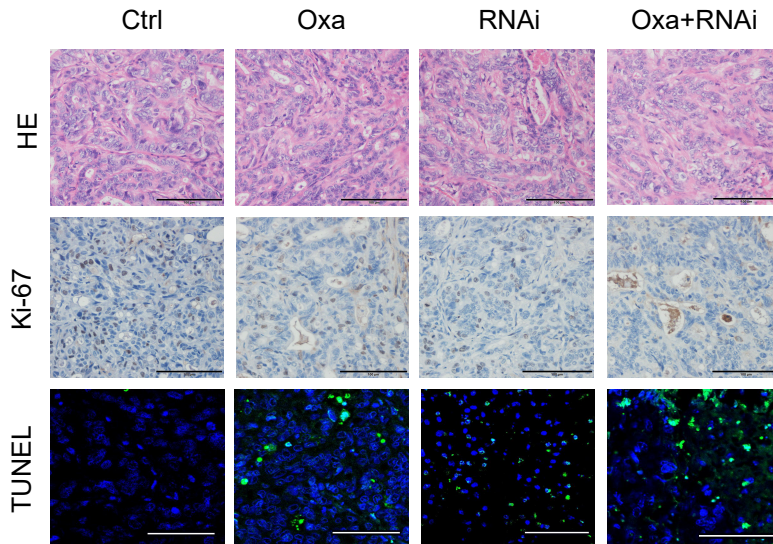

**F**

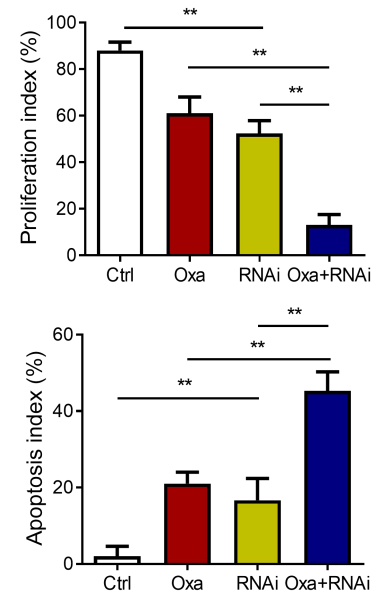

Supplement: Supplementary file 10 — Additional file 10: Figure S5, related to Fig. 5. In vivo experiments elucidated the effect of the inhibition of LINRIS in CRC. (A) qPCR detection shows the relative RNA levels of LINRIS in tumors in two PDX experiments. Error bars, SD of four independent experiments. *P < 0.05 or **P < 0.01 versus the control. (B) Curves of the weights of mice treated with RNAi targeting LINRIS in two PDX experiments. (C and D) The volume growth curves of tumors (C) and the tumor weights (D) of PDX#3 are shown. Ctrl, control. Oxa, oxaliplatin. Error bars, SD of five independent experiments. *P < 0.05 or **P < 0.01 versus the control. (E) Representative images of H&E staining, immunohistochemistry staining of Ki-67 and TUNEL from the tumor sections. Scale bar, 100 μm. (F) Quantification of the proliferation index (Ki-67 proportion) and apoptotic index (TUNEL proportion) in the tumor sections. *P < 0.05, **P < 0.01. [file 12943_2019_1105_MOESM10_ESM.pdf]

Figure S6

A

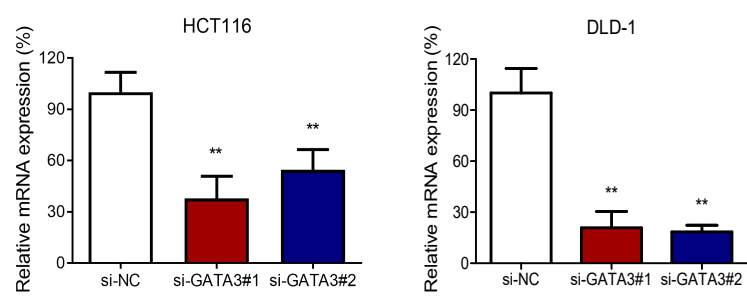

Supplement: Supplementary file 11 — Additional file 11: Figure S6, related to Fig. 6. LINRIS could be inhibited by GATA3 in CRC. (A) qPCR detection shows the inhibition of GATA3 by siRNA in the indicated cells. The data are shown as the mean ± SD; n = 3 independent experiments, two-tailed Student’s t-test, *P < 0.05, **P < 0.01. [file 12943_2019_1105_MOESM11_ESM.pdf]
