# Supplementary material for: LncRNA LINRIS stabilizes IGF2BP2 and promotes the aerobic glycolysis in colorectal cancer
Source: Mol Cancer. 2019 Dec 2;18:174. doi: 10.1186/s12943-019-1105-0 (PMC6886219; doi:10.1186/s12943-019-1105-0)
Supplement: Supplementary file 5 — Additional file 5: Table S3. Correlation between LINRIS expression and clinicopathological features in 118 CRC patients. [file 12943_2019_1105_MOESM5_ESM.docx]

**Table S3** Correlation between *LINRIS* expression and clinicopathological features in 118 CRC patients.

| **Characteristics** | **Low expression**  **n = 58** | **High expression**  **n = 60** | ***P* value** |
| --- | --- | --- | --- |
| **Age** |  |  |  |
| <60 | 33(55.9%) | 26(44.1%) | 0.197 |
| ≥60 | 25(42.4%) | 34(57.6%) |  |
| **Gender** |  |  |  |
| Male | 37(48.1%) | 40(51.9%) | 0.874 |
| Female | 21(51.2%) | 20(48.8%) |  |
| **Differentiation status** |  |  |  |
| Well/Moderate | 40(46.0%) | 47(54.0%) | 0.298 |
| Poor and others | 18(58.1%) | 13(41.9%) |  |
| **Tumor depth** |  |  |  |
| m/sm/mp | 8(44.4%) | 10(55.6%) | 0.799 |
| ss/se/si | 50(50.0%) | 50(50.0%) |  |
| **Lymph node invasion** |  |  |  |
| Absent | 20(47.6%) | 22(52.4%) | 0.849 |
| Present | 38(50.0%) | 38(50.0%) |  |
| **Vascular invasion** |  |  |  |
| Absent | 52(49.5%) | 53(50.5%) | 1.000 |
| Present | 6(46.2%) | 7(53.8%) |  |
| **Distant metastasis** |  |  |  |
| Absent | 44(50%) | 44(50%) | 0.843 |
| Present | 14(46.7%) | 16(53.3%) |  |
| **Clinical stage** |  |  |  |
| I,II | 10(50%) | 10(50%) | 1.000 |
| III,IV | 48(49.0%) | 50(51.0%) |  |

Abbreviations: m: tumor invasion of mucosa; sm: submucosa; mp: muscularis propria; ss: subserosa; se: serosa penetration; si: invasion to adjacent structures.
