# Supplementary material for: LncRNA LINRIS stabilizes IGF2BP2 and promotes the aerobic glycolysis in colorectal cancer
Source: Mol Cancer. 2019 Dec 2;18:174. doi: 10.1186/s12943-019-1105-0 (PMC6886219; doi:10.1186/s12943-019-1105-0)
Supplement: Supplementary file 6 — Additional file 6: Table S4. Effect of factors on OS in the CRC patients in the univariate and multivariate Cox regression model. [file 12943_2019_1105_MOESM6_ESM.docx]

**Table S4** Effect of factors on overall survival in the CRC patients in the univariate and multivariate cox regression model.

| **Factors** | **Univariate** | | **Multivariate** | |
| --- | --- | --- | --- | --- |
|  | **HR (95% CI)** | ***P* value** | **HR (95% CI)** | ***P* value** |
| Age | 1.9568  (1.1109-3.4469) | 0.0233 | 1.6282  (0.895-2.9619) | 0.1122 |
| Gender | 0.5903  (0.3301-1.0553) | 0.1000 |  |  |
| Differentiation status | 1.5559  (0.7911-3.0598) | 0.1520 |  |  |
| Tumor depth | 10.8963  (5.2839-22.4702) | 0.0030 | 4.0654  (0.5294-31.2189) | 0.1797 |
| Lymph node invasion | 3.0959  (1.7441-5.4953) | 0.0012 | 2.4030  (1.1521-5.0124) | 0.0200 |
| Vascular invasion | 1.5064  (0.5910-3.8391) | 0.3129 |  |  |
| Distant metastasis | 5.0513  (2.4545-10.3953) | < 0.0001 | 4.6591  (2.5841-8.4002) | <0.0001 |
| Clinical Stage | 3.5559  (1.7330-7.2965) | 0.0229 | 1.9064  (0.5558-6.5386) | 0.3074 |
| *LINRIS* | 2.0212  (1.1470-3.5617) | 0.0172 | 2.3982  (1.3017-4.4183) | 0.0052 |
