# Supplementary material for: LncRNA LINRIS stabilizes IGF2BP2 and promotes the aerobic glycolysis in colorectal cancer
Source: Mol Cancer. 2019 Dec 2;18:174. doi: 10.1186/s12943-019-1105-0 (PMC6886219; doi:10.1186/s12943-019-1105-0)
Supplement: Supplementary file 12 — Additional file 12: Table S5. Correlation between IGF2BP2 expression and clinicopathological features in 220 CRC patients. [file 12943_2019_1105_MOESM12_ESM.docx]

**Table S5** Correlation between IGF2BP2 expression and clinicopathological features in 220 CRC patients.

| **Characteristics** | **Low expression**  **n = 118** | **High expression**  **n = 102** | ***P* value** |
| --- | --- | --- | --- |
| **Age** |  |  |  |
| <60 | 70 (53.8%) | 60 (46.2%) | 1.000 |
| ≥60 | 48 (53.3%) | 42 (46.7%) |  |
| **Gender** |  |  |  |
| Male | 67 (53.2%) | 59 (46.8%) | 0.892 |
| Female | 51 (54.3%) | 43 (45.7%) |  |
| **Differentiation status** |  |  |  |
| Well/Moderate | 88 (54.7%) | 73 (45.3%) | 0.649 |
| Poor and others | 30 (50.8%) | 29 (49.2%) |  |
| **Tumor depth** |  |  |  |
| m/sm/mp | 4 (44.4%) | 5 (55.6%) | 0.736 |
| ss/se/si | 114 (54.0%) | 97 (46.0%) |  |
| **Lymph node invasion** |  |  |  |
| Absent | 69 (60.5%) | 45 (39.5%) | 0.042 |
| Present | 49 (46.2%) | 57 (53.8%) |  |
| **Vascular invasion** |  |  |  |
| Absent | 103 (52.8%) | 92 (47.2%) |  |
| Present | 15 (60.0%) | 10 (40.0%) |  |
| **Distant metastasis** |  |  |  |
| Absent | 97 (59.5%) | 66 (40.5%) | 0.003 |
| Present | 21 (36.8%) | 36 (63.2%) |  |
| **Clinical stage** |  |  |  |
| I,II | 70 (59.8%) | 47 (40.2%) | 0.058 |
| III,IV | 48 (46.6%) | 55 (53.4%) |  |

Abbreviations: m: tumor invasion of mucosa; sm: submucosa; mp: muscularis propria; ss: subserosa; se: serosa penetration; si: invasion to adjacent structures.
