# Supplementary material for: LncRNA LINRIS stabilizes IGF2BP2 and promotes the aerobic glycolysis in colorectal cancer
Source: Mol Cancer. 2019 Dec 2;18:174. doi: 10.1186/s12943-019-1105-0 (PMC6886219; doi:10.1186/s12943-019-1105-0)
Supplement: Supplementary file 13 — Additional file 13: Table S6. Correlation between LINRIS/IGF2BP2 expression and clinicopathological features in 220 CRC patients. [file 12943_2019_1105_MOESM13_ESM.docx]

**Table S6** Correlation between *LINRIS*/IGF2BP2 expression and clinicopathological features in 220 CRC patients.

| **Characteristics** | ***LINRIS*/IGF2BP2-low**  **n = 76** | **Intermediate**  **n = 68** | ***LINRIS*/IGF2BP2-high**  **n = 76** | ***P* value** |
| --- | --- | --- | --- | --- |
| **Age** |  |  |  |  |
| <60 | 47 (36.2%) | 41 (31.5%) | 42 (32.3%) | 0.702 |
| ≥60 | 29 (32.2%) | 27 (30.0%) | 34 (37.8%) |  |
| **Gender** |  |  |  |  |
| Male | 41 (32.5%) | 39 (31.0%) | 46 (36.5%) | 0.715 |
| Female | 35 (37.2%) | 29 (30.9%) | 30 (31.9%) |  |
| **Differentiation status** |  |  |  |  |
| Well/Moderate | 56 (34.8%) | 53 (32.9%) | 52 (32.3%) | 0.432 |
| Poor and others | 20 (33.9%) | 15 (25.4%) | 24 (40.7%) |  |
| **Tumor depth** |  |  |  |  |
| m/sm/mp | 1 (11.1%) | 5 (55.6%) | 3 (33.3%) | 0.197 |
| ss/se/si | 75 (35.5%) | 63 (29.9%) | 73 (34.6%) |  |
| **Lymph node invasion** |  |  |  |  |
| Absent | 43 (37.7%) | 33 (28.9%) | 38 (33.3%) | 0.567 |
| Present | 33 (31.1%) | 35 (33.0%) | 38 (35.8%) |  |
| **Vascular invasion** |  |  |  |  |
| Absent | 68 (34.9%) | 60 (30.8%) | 67 (34.4%) | 1.000 |
| Present | 8 (32.0%) | 8 (32.0%) | 9 (36.0%) |  |
| **Distant metastasis** |  |  |  |  |
| Absent | 63 (38.7%) | 52 (31.9%) | 48 (29.4%) | 0.019 |
| Present | 13 (22.8%) | 16 (28.1%) | 28 (49.1%) |  |
| **Clinical stage** |  |  |  |  |
| I,II | 43 (36.8%) | 35 (29.9%) | 39 (33.3%) | 0.764 |
| III,IV | 33 (32.0%) | 33 (32.0%) | 37 (35.9%) |  |

Abbreviations: m: tumor invasion of mucosa; sm: submucosa; mp: muscularis propria; ss: subserosa; se: serosa penetration; si: invasion to adjacent structures.
